# Nonalcoholic Fatty Liver Disease (NAFLD). Mitochondria as Players and Targets of Therapies?

**DOI:** 10.3390/ijms22105375

**Published:** 2021-05-20

**Authors:** Agostino Di Ciaula, Salvatore Passarella, Harshitha Shanmugam, Marica Noviello, Leonilde Bonfrate, David Q.-H. Wang, Piero Portincasa

**Affiliations:** 1 Department of Biomedical Sciences & Human Oncology, Clinica Medica “A. Murri”, University of Bari Medical School, 70124 Bari, Italy; agostinodiciaula@tiscali.it (A.D.C.); harshithashanmugam993@gmail.com (H.S.); marica.noviello@outlook.it (M.N.); leonilde.bonfrate@uniba.it (L.B.); 2 School of Medicine, University of Bari Medical School, 70124 Bari, Italy; 3 Department of Medicine and Genetics, Division of Gastroenterology and Liver Diseases, Marion Bessin Liver Research Center, Einstein-Mount Sinai Diabetes Research Center, Albert Einstein College of Medicine, Bronx, NY 10461, USA; david.wang@einsteinmed.org

**Keywords:** lipotoxicity, liver steatosis, mitochondria, nitrosative stress, oxidative stress, steatohepatitis

## Abstract

Nonalcoholic fatty liver disease (NAFLD) is the most common chronic liver disease and represents the hepatic expression of several metabolic abnormalities of high epidemiologic relevance. Fat accumulation in the hepatocytes results in cellular fragility and risk of progression toward necroinflammation, i.e., nonalcoholic steatohepatitis (NASH), fibrosis, cirrhosis, and eventually hepatocellular carcinoma. Several pathways contribute to fat accumulation and damage in the liver and can also involve the mitochondria, whose functional integrity is essential to maintain liver bioenergetics. In NAFLD/NASH, both structural and functional mitochondrial abnormalities occur and can involve mitochondrial electron transport chain, decreased mitochondrial β-oxidation of free fatty acids, excessive generation of reactive oxygen species, and lipid peroxidation. NASH is a major target of therapy, but there is no established single or combined treatment so far. Notably, translational and clinical studies point to mitochondria as future therapeutic targets in NAFLD since the prevention of mitochondrial damage could improve liver bioenergetics.

## 1. Introduction

The liver plays a key role in lipid homeostasis, which includes synthesis, oxidation, and transport of free fatty acids (FFA), triglycerides (TG), cholesterol, and bile acids (BA). Excess accumulation of fat in the liver encompasses several conditions involved in the onset and progression of hepatic steatosis. In particular, the term nonalcoholic fatty liver disease (NAFLD) refers to hepatic steatosis due to unknown causes, encompassing a spectrum of conditions ranging from simple steatosis (nonalcoholic fatty liver) to nonalcoholic steatohepatitis (NASH), cryptogenic cirrhosis, and hepatocellular carcinoma (HCC). The definition of NAFLD rules out heavy alcohol consumption, B and C viruses, several drugs, Wilson’s disease, and starvation as a primary cause. In this context, the term NAFLD describes essentially a metabolic dysfunction-associated fatty liver disease (MAFLD) where hepatic steatosis is associated with at least one of the following three comorbidities: overweight/obesity (especially the expansion of visceral fat), presence of type 2 diabetes mellitus, evidence of metabolic dysregulation [1]. Additional factors contributing to NAFLD include the environment, gut microbiome, deranged glucose-lipid metabolic pathways, metabolic inflammation primarily mediated by innate immune signaling, adipocytokine impairment (e.g., tumor necrosis factor (TNF)-α, adiponectin, resistin, leptin, angiotensin II), and comorbidities [2,3,4].

Inherited predisposition might be implicated in the development and progression of liver steatosis, as underscored by studies showing greater concordance between monozygotic compared to dizygotic twins [5,6]. Although the ultimate application of genetic findings requires additional evidence in clinical medicine, few genetic variants are being investigated so far.

- Adiponutrin patatin-like phospholipase domain-containing protein 3 (PNPLA3) is expressed on the surface of intrahepatocyte lipid droplets and has lipase or lysophosphatidic acyltransferase activity. Carriers of the variant p.I148M have an increased risk of developing NAFLD [7], liver fibrosis and cirrhosis [8], and hepatocellular carcinoma (HCC) [9,10,11];
- Membrane-bound O-acyltransferase domain-containing 7 gene (MBOAT7) has lysophosphatidylinositol acyltransferase activity with the regulation of arachidonic acid levels and shows anti-inflammatory activity. Carriers of the variant rs641738 C > T display deranged MBOAT7 activity [12];
- Transmembrane 6 superfamily member 2 gene (TM6SF2) is involved in hepatic VLDL secretion. Carriers of the variant p.E167K show decreased circulating VLDL and increased liver steatosis [13];
- Glucokinase regulatory protein gene (GCKR) variant p. P446L [14];
- Missense variant in the mitochondrial amidoxime reducing component 1 (MARC1) might have protective effects in NAFLD [15];
- Hydroxysteroid 17-beta dehydrogenase 13 (HSD17B13). The genetic variant is associated with serum levels of alanine aminotransferase (ALT) and aspartate aminotransferase (AST). The rs72613567:TA variant confers a reduced risk of nonalcoholic steatohepatitis (not steatosis) in human liver samples [16].

The role of mitochondrial function in the liver is also being actively investigated in health and disease. Several studies show that deranged mitochondrial function can contribute to fat accumulation and damage in the liver by increased production of reactive oxygen species (ROS), oxidative stress, and defective bioenergetics. These steps likely contribute to the progression of liver disease from NAFL to NASH by mechanisms involving hepatic inflammation, necrosis, and fibrosis.

In this review, we discuss the major pathophysiological mechanisms implicated in NAFLD and focus on the role of mitochondrial dysfunction. We also review current therapeutic approaches in NAFLD with emphasis on mitochondria as potential targets of therapies.

## 2. Physiological Homeostasis of Free Fatty Acids (FFA) in the Hepatocyte

FFA are long-chain carboxylic acids (either saturated or unsaturated). They either derive from the hydrolysis of fat or are synthesized from two carbon units (acetyl-CoA) in the liver, mammary gland, and, to a lesser extent, in the adipose tissue. FFA, also known as non-esterified fatty acids (NEFA), represents the form in which the stored body fat is transported from the adipose tissue to the sites of use. FFA are stored mainly as triglycerides (TG) or in cholesteryl esters and phospholipids. The enzymes lipoprotein lipase and hepatic lipase hydrolyze TG to FFA and glycerol, and then FFA circulate primarily in association with albumin and play a key role in providing energy to the body, especially during fasting. FFA increase in the blood of subjects with central obesity, insulin resistance, and type 2 diabetes.

**Figure 1:**
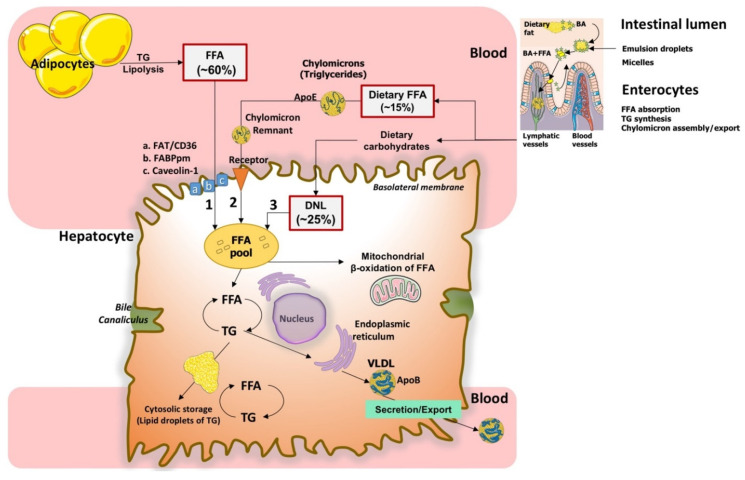
How hepatocytes can provide and metabolize fatty acids. Three major routes provide free fatty acids (FFA) to the liver: (1) circulating FFA (about 60%) made from lipolysis of triglycerides (TG) in adipose tissue [17] can enter the hepatocyte by using specific transporters, (a) the FFA translocase/CD36 transporter, (b) the fatty acid-binding protein (FABP), (c) caveolin-1; (2) dietary FFA (about 15%) contained in TG within ApoE-enriched chylomicrons. These are assembled in the enterocyte following dietary fat digestion in the intestinal lumen by emulsion and micellization with bile acids (BA). In the hepatocyte, chylomicron remnants bind specific membrane receptors with high affinity for the surface protein ApoE; (3) FFA originating from de novo lipogenesis (DNL) (~25%) made mainly from dietary carbohydrates in the hepatocyte. In the hepatocyte, FFA undergo re-esterification with glycerol to form TG stored in small amounts as lipid droplets (less than 5% of cell content). The two major routes of elimination of TG are β-oxidation of FFA in mitochondria and export to blood within very-low-density lipoproteins (VLDL) assembled in the endoplasmic reticulum. In this case, apolipoprotein B (ApoB) undergoes disulfide bond formation and association with TGs by protein disulfide isomerase and microsome triglyceride transfer protein (MTP) at the Golgi apparatus [18]. Abbreviations: BA, bile acids; CD36, fatty acid translocase; DNL, de novo lipogenesis; FABP, fatty acid-binding protein; FFA, free fatty acids; TG, triglycerides VLDL, very-low-density lipoproteins.

Physiologically, the liver accumulates FFA from three different sources: uptake of circulating FFA, de novo synthesis of FFA, and uptake of dietary FFA (Figure 1) [17].

### 2.1. Uptake of Circulating FFA

About 60% of total FFA pool in the liver derives from the uptake of FFA products of lipolysis of TG in adipocytes, which occurs under insulin control involving three lipases: the adipose tissue lipase (ATGL), the hormone-sensitive lipase (HSL), and the monoglyceride lipase, co-lipases, and lipid droplet proteins [19,20,21]. FFA are usually bound to plasma albumin. Uptake of FFA by the liver involves diffusion across phospholipid bilayers and transport mediated by transmembrane transporters, namely plasma membrane FFA binding protein (FABPpm), fatty acid transporter protein (FATP), caveolins, fatty acid translocase (FAT)/CD36 [22]. As shown in Figure 1, in the hepatocyte, FFA undergo re-esterification with glycerol to form TG are stored as lipid droplets in small amounts (less than 5% of cell content). The two major routes of elimination of TG are β-oxidation of FFA in mitochondria or formation/export (as TG) of very-low-density lipoproteins (VLDL) assembled in the endoplasmic reticulum and exported to blood. Notably, hyperinsulinemia increases intracellular fat accumulation by downregulating microsomal triglyceride transfer protein (MTP) gene expression and upregulating VLDL degradation in hepatocytes [18]. In the liver cytosol, FFA are transformed into fatty acyl-CoA via acyl-CoA synthase.

### 2.2. De Novo Lipogenesis

About 25% of total FFA pool in the liver originates from dietary sugars (excess glucose and fructose) during the process of de novo lipogenesis (DNL) through acetyl-CoA, in which mitochondria play a major role (see below). Insulin mediates both the transport of absorbed dietary carbohydrates in the cells and their storage as glycogen in the skeletal muscle and the liver. Due to the absence of the glucose-6-phosphate phosphatase in the muscle, glycogen will be used as the main energy source in anaerobic glycolysis, whereas in the liver, it will be used to maintain the correct glycemia.

Hepatic DNL is responsive to insulin, especially after a high-carbohydrate meal. Enzymes responsible for hepatic lipogenesis are the sterol regulatory element-binding protein-1c (SREBP-1c), sensitive to insulin via a phosphoinositide 3-kinase (PI3K)-dependent mechanism and the liver X receptor α (LXRα). This, in turn, promotes the expression of *SREBP-1c*, its target genes fatty acid synthase (*FAS*), acetyl-CoA carboxylase (*ACC*), stearoyl-CoA desaturase (*SCD1*), and lipin [23]. Carbohydrate-responsive element-binding protein (ChREBP) is directly activated by glucose and not by insulin. DNL is one pathway eventually involved in NAFLD [17].

### 2.3. Uptake of Dietary FFA

About 15% of total FFA pool in the liver originates from the uptake of dietary FFA [24] (Figure 1). In the enterocytes, TG are re-synthetized and incorporated into nascent chylomicrons with cholesteryl esters, phospholipids, and apolipoprotein ApoB-48. Chylomicrons are then transported into lymph (exocytosis) and blood, where the serum lipoprotein lipase hydrolyzes TG to FFA and glycerol. Ultimately, FFA are taken up by the adipose tissue and liver [25]. BA also acts as potent metabolic regulators in the terminal ileum via activation of the nuclear orphan farnesoid X receptor (FXR) plus pregnane X receptor (PXR) and the membrane-associated G-protein-coupled bile acid receptor-1 (GPBAR-1), with target organs being the liver and muscle, adipocytes and brown adipose tissue for energy expenditure [26,27]. See also our recent reviews on this topic [2,28].

As depicted in Figure 1, in the hepatocyte, FFA undergo re-esterification with glycerol to form TG, which are stored in lipid droplets in small amounts (less than 5% of cell content). Lipid droplets are complex bodies made of neutral lipids (TG, diacylglycerol, cholesterol esters, and retinol esters). A phospholipid monolayer surrounds the droplet, contains free cholesterol, and is embedded with a diverse group of proteins and enzymes [29]. Overall, this structure governs the formation of the droplet, the synthesis and hydrolysis of its lipids, and the movement of lipids to specific intracellular and secretory pathways. In the adipocyte, lipid droplets can be considered as an energy store. In the liver, muscle, and heart, the droplets provide local energy supply (membrane biogenesis and repair). Hydrolysis of the TG from lipid droplets releases FFA ligands used for transcription factors. Cholesteryl esters or retinyl esters may act as mediators of signaling and inflammation, as substrates for steroid hormone biosynthesis in cells of the adrenal cortex, testes, and ovaries, and as substrates for surfactant synthesis in type II alveolar pneumocytes. Stored TG in hepatocytes represent the pool of substrate for VLDL biogenesis as well as for β-oxidation [30].

## 3. Mitochondrial Function in the Hepatocyte

**Figure 2:**
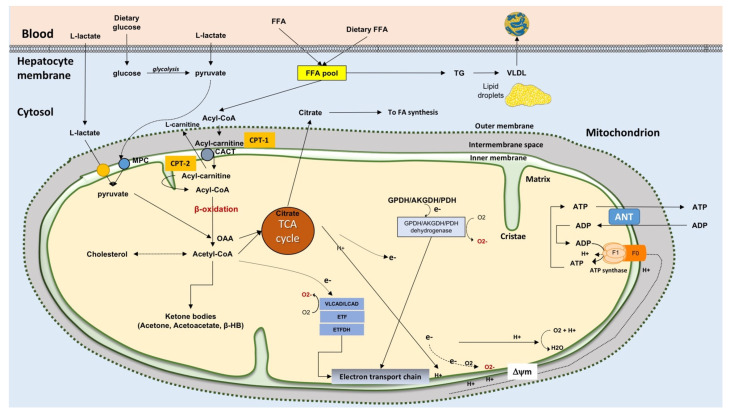
Mitochondrial adaptation and oxidative stress in NAFLD. Mitochondrial oxidative metabolism and hepatocyte energy homeostasis depend on FFA β-oxidation, the tricarboxylic acid cycle (TCA), electron flow along the electron transport chain, electrochemical proton gradient generation, and ATP synthesis. When β-oxidation is impaired (e.g., in liver steatosis), lipotoxic lipids accumulate. Moreover, dysfunction of the electron transfer chain can result in ROS generation. ROS are generated from glycerol 3-phosphate dehydrogenase (GPDH), pyruvate dehydrogenase (PDH), and α-ketoglutarate dehydrogenase (AKGDH) as minor contributors. In starvation, ketone bodies are produced due to the absence of oxaloacetate used in gluconeogenesis. Dietary carbohydrates and dietary FFA are the two major sources contributing to the FFA pool in the hepatocyte. When fatty acid synthesis occurs, glucose essentially from dietary sources is converted to pyruvate during glycolysis. Pyruvate can enter the mitochondrion via the mitochondrial pyruvate carrier (MPC) as well as can be synthesized from L-lactate after transport of L-Lactate in the matrix, via its own carrier, and oxidation via the mitochondrial L-lactate dehydrogenase [35,36]. In the matrix, pyruvate can provide Acetyl-CoA via the pyruvate dehydrogenase complex and oxaloacetate (OAA) via the pyruvate carboxylase. Due to citrate synthase, pyruvate and oxaloacetate provide citrate that can be exported to allow for FFA synthesis in the cytoplasm in the de novo lipogenesis (DNL). How citrate can be exported outside mitochondria is described below. Abbreviations: ACC, acetyl-CoA carboxylase (ACC); ANT, adenine nucleotide translocator; CACT, carnitine-Acylcarnitine Transferase; CPT-1, carnitine palmitoyl-transferase-1; CPT-2, carnitine palmitoyl-transferase-2; DNL, de novo lipogenesis; electron transfer flavoprotein (ETF); ETFDH, ETF dehydrogenase; FFA, free fatty acids; β-HB, β-hydroxybutyrate; MPC, mitochondrial pyruvate carrier; OAA, oxaloacetate; PEP, phosphoenolpyruvate; TG, triglycerides; VLDL, very-low-density lipoprotein [37].

Liver mitochondria (~500–4000 per cell [31], occupying about 18% of the entire cell volume [32]) play a key role in the metabolic pathways and signaling networks [33] (see Figure 2). Mitochondria generate ATP by using as energy source substrates derived from the catabolism of carbohydrates, lipids, and proteins.

The outer mitochondrial membrane contains numerous integral proteins (porins) that make the membrane permeable to molecules up to 5000 Daltons. The inner membrane is impermeable to both ions and molecules that require specific membrane transporters to enter or exit the matrix. The four types of proteins in the inner mitochondrial membrane include the respiratory chain, the ATP synthase, the specific proteins allow for transport of metabolites, ions, vitamins/vitamin derivatives into and from the matrix, and protein import machinery. Hundreds of enzymes are contained in the matrix where pyruvate and FFA are oxidized, almost all the citric acid cycle reactions, some reactions of urea cycle take place; also, reactions of the amino acid metabolism (in particular those catalyzed by glutamate dehydrogenase, glutaminase, and by some aminotransferase occurs in the matrix. Studies described the mitochondrial pyruvate kinase [34]. The nuclear DNA encodes about 90% mitochondrial proteins, while the others are encoded by the mitochondrial DNA (mtDNA), a circular double-stranded molecule located in the mitochondrial matrix. Importantly, the oxidative damage can severely impair the mtDNA function [32].

In fatty acid catabolism, in the hepatocyte cytosol, FFA are transformed into fatty acyl-CoA via acyl-CoA synthase (Figure 2). Acyl-CoA + carnitine in a reaction catalyzed by carnitine palmitoyl-transferase 1, (CPT-1) located in the outer side of the inner mitochondrial membrane, gives CoA and acylcarnitine. Acylcarnitine can then enter mitochondria in exchange with L-carnitine via the acylcarnitine/L-carnitine antiporter. Due to CPT-2 localized at the matrix side of the inner membrane, L-carnitine is released, and the acyl-CoA is oxidized via the β-oxidation to acetyl-CoA. Final oxidation of acetyl-CoA to carbon dioxide and water occurs in the tricarboxylic acid (TCA) cycle and the electron transport chain with ATP production. In lipid synthesis, FFA in the hepatocyte cytosol is esterified with glycerol to form TG via the key enzymes, diglyceride acyltransferase (DGAT)1 and DGAT2, and exported to blood as very-low-density lipoprotein (VLDL) [38]. Conversely, TG can be hydrolyzed by hydrolases, e.g., the patatin-like phospholipase domain-containing protein 3-PNPLA3, also known as adiponectin, and this step contributes to enrich the FFA pool [19,24]. Ketone body synthesis depends on prolonged starvation (or diabetes) when oxaloacetate is depleted due to its involvement in gluconeogenesis. Then, acetyl-CoA does not enter the TCA cycle, and in the mitochondria, is converted to acetone, acetoacetate, and β-hydroxybutyrate, β-HB (ketone bodies).

**Figure 3:**
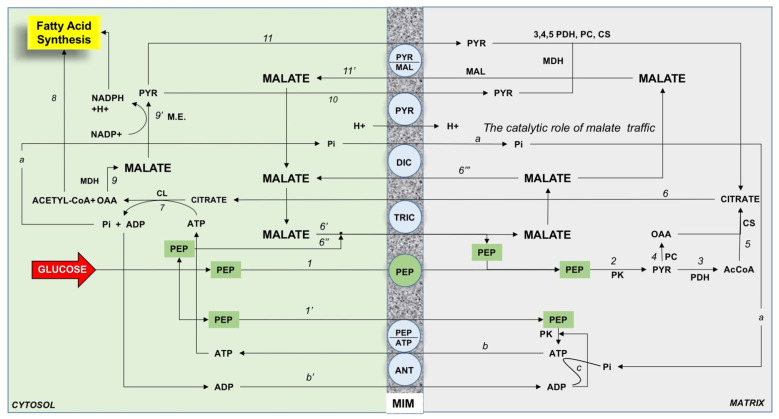
The role of the phosphoenolpyruvate (PEP) dependent mitochondrial traffic in rabbit kidney fatty acid synthesis. The following scenario is proposed: PEP enters mitochondria via the putative PEP carrier (1); inside the matrix PEP produces pyruvate (PYR) via the mitochondrial pyruvate kinase (PK) (2); PYR is both oxidized to acetyl-CoA via the pyruvate dehydrogenase (PDH) (3) and carboxylated to oxaloacetate (OAA) via the pyruvate carboxylase (PC) (4); acetyl-CoA and OAA gives citrate via the citrate synthase (CS) (5); citrate is exported in the cytosol (6) in exchange with malate (6′) and/or PEP (6″); in the cytosol citrate produces OAA and acetyl-CoA via the ATP-citrate lyase (CL) (7); acetyl-CoA is used for fatty acid synthesis (8); OAA is reduced to malate via the cytosolic malate dehydrogenase (9); malate gives NADPH for fatty acid synthesis and PYR via the malic enzyme (M.E.) (9′); PYR enters mitochondria via its own carrier (10) and in exchange with malate via the PYR/malate antiporter (11); malate just exported and that exported via the dicarboxylate carrier in exchange with phosphate formed in the CL reaction (a) promotes further citrate efflux in a catalytic traffic. In this manner, most of the malate formed in (9) is available for NADPH production. ATP formed in PK reaction is exported in the cytosol in exchange with PEP (b) via the putative PEP/ATP antiporter and or in exchange with ADP (b′) to provide further ATP via ATP synthase (c). Legend: MIM, mitochondrial inner membrane.

Mitochondria play a major role in fatty acid synthesis. The main point is that citrate, the precursor of the fatty acid synthesis, is synthesized in the mitochondrial matrix and must be exported outside mitochondria where fatty acid synthesis occurs. How this could occur is described in Figure 3. The scheme is based on a variety of experimental findings obtained by using rabbit kidney mitochondria described in [39]; in particular, the role of phosphoenolpyruvate has been considered after the discovery of the mitochondrial pyruvate kinase in pig liver [34] as a major precursor of citrate synthesis in the mitochondria.

Glucose in the hepatocytes is mainly metabolized to pyruvate via glycolysis and then to acetyl-CoA to generate ATP in the TCA cycle and oxidative phosphorylation. In hypoglycemia, pyruvate, derived from L-lactate and alanine, and other glucose precursors, promote gluconeogenesis. De novo FFA synthesis includes citrate export from mitochondria in a carrier-mediated manner, the ATP-dependent citrate lyase reaction that provides from the cytosol acetyl-CoA and oxaloacetate. Oxaloacetate is reduced to malate via malate dehydrogenase; malate via the malic enzyme reaction provides NADPH used in FFA synthesis and pyruvate that comes back into mitochondria. Acetyl-CoA via acetyl-CoA carboxylase (ACC) (activated by citrate) produces malonyl-CoA to start FFA synthesis [40]. In the cytoplasm, citrate regulates the glycolytic flux by inhibiting the phosphofructokinase, thus favoring the glucose entry in the pentose cycle to provide NADPH for FFA synthesis. To date, the mitochondrial transport that occurs in FFA synthesis is not fully elucidated.

In case of excess FFA influx, hepatic fatty acid β-oxidation is insufficient, and this leads to accumulation of lipotoxic intermediates (see the section on mitochondrial dysfunction): The process of mitochondrial β-oxidation generates NADH and FADH_2_ with electron transport to the electron transport chain (ETC); the rate of electron flow through the ETC is limited by the ATP rate turnover and by the rate of processes that use the electrochemical proton gradient. Impaired electron transfer along the ETC leads to the generation of ROS. Mitochondrial ROS also originate from reactions catalyzed by enzymes such as long acyl-CoA dehydrogenase (LCAD), very long-chain acyl-CoA dehydrogenase (VLCAD), glycerol 3-phosphate dehydrogenase (GPDH), α-ketoglutarate dehydrogenase (AKGDH), and pyruvate dehydrogenase (PDH) [41].

## 4. Epidemiology and Manifestations of NAFLD

Epidemiological studies show that NAFLD has become one of the most popular chronic liver disorders in western countries (10% to 46% of prevalence in the USA [42,43,44]). The median prevalence of NAFLD is about 20% worldwide, with a progressively increasing trend [45]. This is likely due to the increasing prevalence of obesity, type 2 diabetes mellitus, sedentary lifestyles, dyslipidaemia, and metabolic syndrome, mainly in North America and Europe [45,46,47,48]. NAFLD is detected in up to 70% of overweight adults and in more than 90% of morbidly obese [49,50,51]. However, both NAFLD and NASH can also occur in lean subjects [47], and this condition is frequent in Asia [52]. The risk of developing cardiovascular disease [53], premature death [54], and insulin resistance [55] increases as well in NAFLD people. Hepatic steatosis occurs with excess accumulation of TG in the hepatocytes, and the minimum criterion for a histologic diagnosis of NAFLD is >5% steatotic hepatocytes in a liver tissue section [56]. NAFLD is the most frequent type of liver steatosis, while other causes include excessive alcohol intake, viral hepatitis C (in particular genotype 3), lipodystrophy, Wilson disease, starvation, parenteral nutrition, abetalipoproteinemia, hepatotoxic drugs (e.g., methotrexate, tamoxifen, glucocorticoids, amiodarone, valproate, and anti-retroviral agents for HIV), pregnancy, HELLP (hemolytic anemia, elevated liver enzymes, low platelet count) syndrome, Reye syndrome, and inborn errors of metabolism (i.e., lecithin-cholesterol acyltransferase deficiency, cholesterol ester storage disease, Wolman disease). The spectrum of NAFLD is depicted in Table 1.

Although some patients with NAFLD may complain of vague symptoms (i.e., fatigue, malaise, right upper abdominal discomfort), NAFLD remains asymptomatic in most cases. Only a few patients exhibit mildly elevated or fluctuations of liver alanine aminotransferases. NAFLD can be detected by abdominal ultrasonography showing increased liver echogenicity (“bright liver”), computed tomography (decreased hepatic attenuation), or by magnetic resonance imaging (increased fat signal).

## 5. General Features of Diagnosis of NAFLD

The diagnosis of NAFLD relies on liver imaging and histology. Other causes of liver steatosis ad chronic liver diseases must be excluded [63], while alcohol consumption should be absent or very limited, i.e., not more than three standard drinks/day (i.e., 21 drinks/week) in men or not more than two drinks/day (i.e., 14 drinks/week) in women (equal to 14 g of pure alcohol/standard drink = 98 kcal) (see the practice guidance from the American Association for the Study of Liver Diseases (AASLD) [64].

A variety of aspects should be considered for NAFLD management. According to the AASLD guidelines, a systematic screening for NAFLD is not yet advisable. There is no consensus about the true cost-effectiveness of screening [64], and precise characterization of NAFLD populations requires specific research protocols [40]. Definitive diagnostic tests are still lacking for NAFLD. On the other hand, liver biopsy is invasive and cannot be performed routinely. In addition, the standard treatment for NAFLD is missing, apart from healthy lifestyles [46,47]. Contrarily, early identification and targeted treatment of NASH could attenuate the multiple consequences related to progressive liver disease (e.g., economic burden due to health care for end-stage liver disease, need for liver transplantation, and care of patients with HCC). Due to the relevant metabolic links, NAFLD puts the patients at increased risk for extrahepatic complications, i.e., cardiovascular disease and malignancy [65,66].

## 6. Lipotoxicity during Insulin Resistance and the Onset of Liver Steatosis

The events that influence the above-mentioned pathways of FFA homeostasis in the hepatocyte can contribute to the development of NAFLD (Table 2).

**Figure 4:**
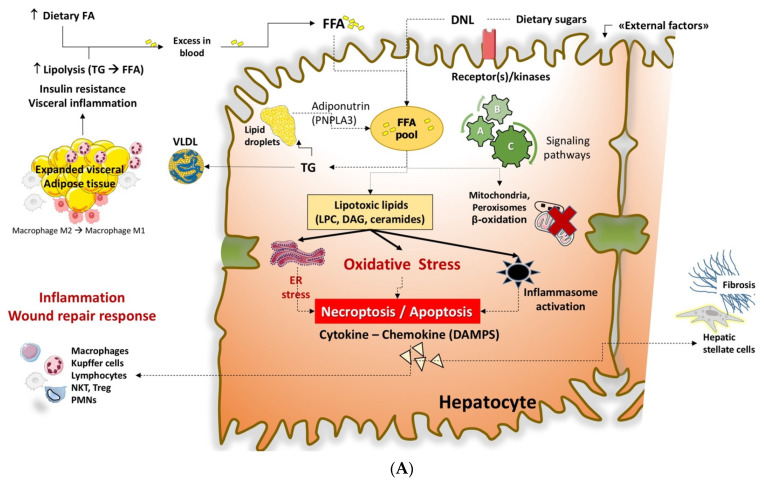

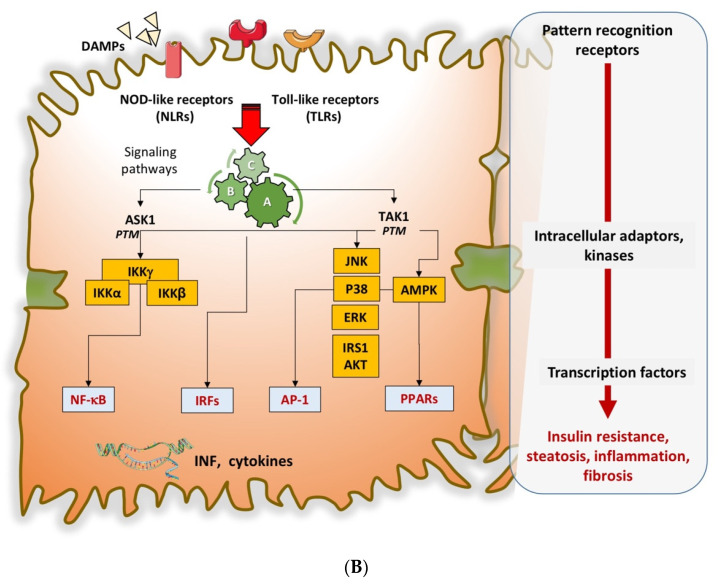
(**A**) Mechanisms of lipotoxicity in the liver contributing to onset and progression of NAFLD. The “lean” adipose tissue express anti-inflammatory cytokines (i.e., adiponectin, interleukin IL-4, IL-10, IL-13, Transforming growth factor (TGF)-β, nitric oxide (NO)), which can both activate the M2 macrophagic response and inhibit the neutrophil-mediated inflammation. The expanded hypertrophic (and apoptotic) visceral adipose tissue (i.e., obesity) is associated with the secretion of proinflammatory molecules such as leptin, resistin, IL-6, and tumor necrosis factor (TNF)-α, that can activate an M1 macrophage-response [67]. These steps result in insulin resistance, a chronic “metabolic” inflammatory status, and increased lipolysis of TG with excess FFA in blood directed to the liver. In addition, dietary FFA can increase because of dietary modifications. De novo lipogenesis (DNL) from dietary sugars will also contribute to the expansion of the intracellular (hepatocyte) FFA pool. The protein adiponutrin (namely the patatin-like phospholipase domain-containing protein 3, PNLPA3) is involved in lipid droplet lipolysis, which provides FFA enriching the FFA pool. Excessive accumulation of intracellular FFA paves the way to decreased mitochondrial β-oxidation and defective secretion/export of very-low-density lipoproteins (VLDL) to blood, which is enriched with FFA as TG. Thus, lipotoxic species (Lysophosphatidylcholine, LPC; diacylglycerol, DAG; ceramides) can accumulate and mediate endoplasmic reticulum (ER) stress and oxidative stress. Another step includes the activation of the inflammasome, i.e., the multiprotein cytoplasmic complex that responds to damage-associated molecular patterns (DAMPs) as part of the innate immunity response. Additional abnormalities are the dysregulation of adipocytokines, depletion of ATP, production of toxic uric acid, periodic hypoxia (i.e., during sleep apnea in extremely obese patients), and toxic products from the gut microbiome, which include tumor necrosis factor (TNF)-α, endogenous ethanol, and endotoxins such as lipopolysaccharides (LPS). All the above-mentioned conditions promote the NASH phenotype manifesting with hepatocellular injury, inflammation, stellate cell activation, and progressive accumulation of excess extracellular matrix. Intracellular organelles, the nucleus, receptors, and signaling pathways are also targets of ongoing cellular damage. See also [40,66,68,69]. (**B**) Further mechanisms of lipotoxicity in the liver contributing to the onset and progression of NAFLD. The cartoon shows that circulating damage-associated molecular patterns (DAMs) activate the pattern recognition receptors (PRRs), which include the NOD-like receptors (NLRs) and Toll-like receptors (TLRs). This step leads to the activation of signaling pathways and kinases, i.e., apoptosis signal-regulating kinase 1 (ASK1) and TGF-b-activated kinase 1 (TAK1). Post-transcriptional modification (PTM) activates ASK1 and TAK1, and this step leads to the activation of other kinases such as the C-Jun N-terminal kinase (JNK), the AMP-activated kinase, (AMPK), and IkB. Further transcription factors take part in this process, i.e., nuclear factor (NF)-kB, interferon regulatory factors (IRFs), activator protein 1 (AP-1), and peroxisome proliferator-activated receptors (PPARs). This step is followed by the production of inflammatory cytokines and chemokines with metabolic consequences typical of NAFLD, including insulin resistance, steatohepatitis, fibrogenesis, etc. Additional endogenous targets contribute to regulating the innate immune elements playing a role in the necro-inflammatory NASH. Involved are CASP8 and FADD-like apoptosis regulator (CFLAR), tumor necrosis factor (TNF) a-induced protein 3 (TNFAIP3), cylindromatosis (CYLD), transmembrane BAX inhibitor motif-containing 1 (TMBIM1), dual-specificity phosphatase 14 (DUSP14), TNF receptor-associated factor 6 (TRAF6), TRAF1, TRAF3, tripartite motif 8 (TRIM8), dickkopf-3 (DKK3), and TRAF5 (See also [40]).

A set of metabolic abnormalities is able to interfere with the pathway of FFA, which includes insulin resistance, expansion of visceral fat, sedentary behaviors, and a high-calorie diet. Metabolic stress is associated with a chronic inflammatory status and major changes in hepatic lipidology and accumulation of several lipotoxic species. These aspects are schematically described in Figure 4A,B.

## 7. FFA and Toxic Lipids in NAFLD

Overall, the development of liver steatosis is associated with the accumulation of FFA and a variety of toxic lipids.

### 7.1. Free Fatty Acids (FFA)

During insulin resistance and NAFLD, there is an increase in peripheral lipolysis with an intrahepatic influx of FFA, internalized by a system involving CD36, overexpressed in insulin resistance states [70]. Additional abnormalities include (a) activation of DNL in which the ingested glucose is re-directed to the liver [38,71] where FFA synthesis occurs [19,72]); (b) increased influx of dietary FFA; (c) decreased mitochondrial oxidation of FFA; (d) increased assembly and deposition of TG as droplets, and (e) decreased secretion/export of very-low-density lipoproteins (VLDL) that contain 60% TG, 20% cholesterol/cholesteryl esters, 15% phospholipids and 5% proteins (ApoB-100, ApoC, and ApoE) [73]. Excessive FFA synthesis results in the formation of acyl-CoA, which is esterified to produce TG stored in the hepatocytes. Despite all types of FFA may contribute to steatosis, saturated FFA are especially toxic [74], e.g., palmitic acid (C16:0) and stearic acid (C18:0) are more toxic than monounsaturated FFA (e.g., oleic acid, C18:1), which synthesis depends on the enzyme stearoyl-CoA desaturase [75], and contribute to reduced cell death via decreased levels of proapoptotic proteins (BIM (BCL2L11) and PUMA (BBC3)) while promoting the sequestration of palmitic acid in TG [76]. Notably, NASH individuals show more saturated FFA compared to individuals without NAFLD. In addition, the amount of polyunsaturated FFA (PUFA) is progressively lower in accordance with NAFLD severity [77]. Potential protective effects of PUFA are anticipated (see the section on therapeutic agents). The detrimental effect of saturated FFA is now clear since an isocaloric diet enriched in saturated FFA increased liver fat and was associated with postprandial hyperglycemia, whereas an isocaloric diet high in sugar had no effect on liver fat and was associated with only minor metabolic changes [78].

### 7.2. Triglycerides (TG)

As mentioned before, TG can accumulate because of increased delivery of FFA from insulin-resistant adipose tissue, intrahepatic de novo lipogenesis, and dietary fat [17]. The appearance is that of micro- and macro-droplets, which, at least initially, act as a type of inert form with a protective role against the ongoing lipotoxic cell injury [17,73]. In line with this hypothesis, if TG synthesis is blocked via inhibition of diacylglycerol acyltransferase 2, the steatosis decreases but oxidative stress, inflammation, and fibrosis increase [79]. As shown in double knockout mice with simultaneous modulation of FFA oxidation and DNL, the worst scenario would be an accumulation of lipid intermediates and low levels of TG in generating oxidative stress, inflammation, and cell damage [80]. Some proteins binding lipid droplets, e.g., perilipin-5, can also play a role [81,82] since mice with defective perilipin-5 exhibited smaller sizes of lipid droplets and increased lipolysis and lipotoxicity [83].

### 7.3. Lysophosphatidylcholine (LPC)

LPC originates in the cell from phosphatidylcholine via phospholipase A2 and from the extracellular phase via lecithin-cholesterol acyltransferase. Animal models and NASH individuals exhibit increased LPC [84]. LPC mediates intracellular damage such as endoplasmic reticulum (ER) stress, activation of apoptotic pathways downstream of JNK, and also interacts with palmitate [84,85].

### 7.4. Ceramides

Ceramides belong to the sphingolipid metabolism and originate from serine or alanine or glycine plus palmitate (or myristate, stearate) (enzyme: serine palmitoyl-transferase) or from sphingomyelin (enzyme: neutral sphingomyelinase) in the endoplasmic reticulum, in the plasma membranes, in lysosomes and mitochondria. Ceramides function as either intra- or intercellular messengers and as regulatory molecules and play roles in signal transduction, inflammation, angiogenesis, insulin resistance, neurodegeneration, and cancer/cancer therapy. Ceramides can interact with tumor necrosis factor (TNF)-α, interleukin-1 (IL-1), and IL-6 during inflammation and steps involving cell toxicity [85]. As a consequence, in animal models of NAFLD, the inhibition of ceramide synthesis is associated with decreased liver steatosis, cell injury, and insulin sensitivity [86,87]. In NASH, ceramides promote the release of extracellular vesicles (EV) involved in cell-cell communication.

### 7.5. Free Cholesterol

Mounting evidence points to a role for free cholesterol in the pathogenesis of NAFLD manifestations. Free cholesterol is incorporated within the phospholipid monolayer surrounding the triglyceride lipid droplets [30]. Properties related to drop fusion and size can be influenced by free cholesterol [29]. Free cholesterol likely plays a role in inflammation, fibrosis, and liver injury during NASH [88]. Target cells are hepatocytes, stellate, and Kupffer cells. A putative mechanism in the sequence liver steatosis/NASH includes increased expression of sterol regulatory element-binding protein (SREBP)-2, upregulation of 3-hydroxy-3-methyl-glutaryl-coenzyme A (HMGCoA) reductase, increased synthesis of free cholesterol in the mitochondria [89], apoptosis, and JNK-dependent proinflammatory pathways. Further studies need to explore the role of cholesterol crystallization in NASH, starting from the periphery of large lipid droplets and initiating the activation of NLRP3 inflammasome and release of proinflammatory cytokine IL-1β, pyroptotic cell death, Kupffer cell aggregation, migration of activated macrophages and neutrophils, and activation of stellate cells with a propensity to liver fibrosis [88].

Increased production of lipotoxic species in NAFLD/NASH leads to hallmarks of cellular events [90]. These include insulin resistance, liver steatosis, oxidative stress, mitochondrial dysfunction, ER stress, inflammation, apoptosis, and fibrosis [40,90]. In particular, the ER stress contributes to the production of ROS, thus leading to cell death and the release of damage-associated molecular patterns (DAMPs).

In more detail, the mechanism of lipotoxicity (Figure 4A,B) involves receptor/kinase-mediated interactions and signaling pathways; the ER and other intracellular organelles, including mitochondria and the nucleus, play a significant role [91]. Further consequences are ER stress, inflammatory changes such as metabolic inflammation or meta-inflammation [92], production of ROS, and cell death with DAMPs [93]. The innate immune system becomes part of this metabolic inflammation, with the recruitment of Kupffer cells, dendritic cells, lymphocytes, as well as hepatocytes and endothelial cells [94,95]. With the activation of transcription factors, other events include the release of inflammatory cytokines and chemokines, stimulation of hepatic stellate cells with collagen deposition, and further aggravation of the insulin resistance status. All these steps are harmful features in the progression of NASH [96].

Other targets of lipotoxicity are adipose tissue, skeletal muscle, heart, pancreatic islets, brain (certain areas), and intestinal microbiota.

## 8. Mitochondrial Dysfunction in NAFLD and NASH

The efficiency of mitochondria in providing energy to the cell depends on a variety of aspects, including mitochondrial biogenesis (including protein transport from the cytosol, mitochondrial protein synthesis dependent on the mitochondrial DNA and vitamin/vitamin derivative transport and processing, etc.), mitochondrial transport and energy metabolism dependent on a variety of mitochondrial carriers [97] and on the enzyme/complexes located in the different mitochondrial compartments. To investigate whether and how mitochondria are modified in diseases is a hard task, and the difficulty also applies to NAFLD [69]. A review dealing with the role of mitochondria in NAFLD [21] discussed several aspects of this topic, but mechanisms involving the transport of acyl-CoA in the matrix and the role of mitochondria in fatty acid synthesis have not been adequately addressed. Indeed, whether and how mitochondrial disfunction takes place in NAFLD and NASH remains to be established exhaustively. Here, we report several experimental findings dealing with potential mitochondrial dysfunctions occurring in liver steatosis.

### 8.1. FFA Import in Mitochondria, Electron Transfer Chain Efficiency

A modification of the FFA import into mitochondria depends on the oxidation of CPT1 [98].

In a paper aimed at ascertaining both whether FFA transport into the mitochondria is impaired in patients with NASH and to assess the activity of the mitochondrial respiratory chain enzymatic complexes in these patients [99], it was found that the activities of the respiratory chain complexes were decreased in liver tissue of patients with NASH. This dysfunction correlated with serum TNF-a, insulin resistance. No change in the hepatic carnitine content and CPT activity was found in patients with NASH with respect to healthy people, but no investigation was made on the acyl-carnitine/carnitine antiporter, which makes possible FFA transport in mitochondria. Themselves similar data, i.e., data regarding a single enzyme/process, have limited importance because the rate-limiting step of the process leading to the liver pathology remains unknown, thus preventing the identification of a possible therapeutic target.

### 8.2. Diet and Mitochondrial Disfunction with ROS Production

A western type diet results in liver steatosis, as reported in a study dealing with the mitochondrial adaptation in steatotic mice [100]. The association of insulin resistance with mitochondrial abnormalities was described in NAFLD, suggesting that peripheral insulin resistance, increased fatty acid β-oxidation, and hepatic oxidative stress are present in both fatty liver and NASH, but NASH alone is associated with mitochondrial structural defects [101].

The consolidation of liver steatosis decreases the efficiency of the respiratory transport chain with the production of ROS and endoplasmic reticulum stress. ROS are formed if electrons leak out from one of the complexes from the electron transport chain. At this stage, the electrons can interact with oxygen to form superoxide, products that damage mitochondria by peroxidizing mitochondrial DNA [101], phospholipid acyl chains, and enzymes of the respiratory transport chain [74]. In addition, the excessive lipids flow toward the hepatocytes can derange the dephosphorylation capacity of the mitochondrial voltage-dependent anion channel, the inner membrane permeabilization, leading to depolarization of mitochondria, decreased ATP synthesis, loss of antioxidant capacity [102,103,104], excessive ROS generation [105,106], and production of lipid peroxidation products such as malondialdehyde (MDA) and 4-hydroxy-2′-nonenal (HNE) [107]. Further events include inflammation, apoptosis, and liver fibrosis. Saturated FFA can derange the composition of mitochondrial membranes, this favoring the progression of NAFLD [51].

### 8.3. Metabolism Alterations, Lipotoxicity and Apoptosis

Structural abnormalities of mitochondria are disclosed by apoptosis [73] and cristae swelling [108]. Functional changes of the mitochondria also occur during NAFLD [109]. This was shown when challenging the mitochondrial capacity to metabolize ^13^C-ketoisocaproic acid [110] or the medium-chain fatty acid ^13^C-octanoic acid [111] by stable-isotope ^13^C-breath test [112,113,114].

In an investigation made to ascertain whether humans with NAFLD have abnormal in vivo hepatic mitochondrial metabolism [72], it was found that individuals with NAFLD had higher rates of both lipolysis (about 50%) and gluconeogenesis (about 30%), pathways in which mitochondria play a major role. A positive correlation existed between the high intrahepatic TG content and both mitochondrial oxidative and anaplerotic fluxes. How this event occurs remains to be established.

Mitochondria become the target of lipotoxicity, as found in type 2 diabetes mellitus and also in NAFLD [72,115]. With NASH, reshaping of mitochondrial lipids can occur [77], leading to increased mitochondrial mass and respiratory capacity [116].

NAFLD/NASH can promote hyperglycemia via mechanisms involving hepatic oxidative metabolism and gluconeogenesis. A study used five simultaneous stable isotope tracers in 24-h-fasted (ketotic) individuals and up to 50% hepatic TG content [117]. The worsening of hepatic steatosis and glycemia was associated with progressive deterioration of ketogenesis. In NAFLD, the alternative pathway for acetyl-CoA oxidation in the TCA cycle became upregulated as ketone production diminished and correlated positively with rates of gluconeogenesis and plasma glucose concentrations. Likely, the increased respiration and energy generation may explain the increased glucose production and hyperglycemia in NAFLD because of acetyl-CoA metabolization in the liver. Hepatic anaplerotic/cataplerotic pathways contribute to biosynthesis and are energetically backed by increased oxidative metabolism. This pathway can lead to oxidative stress and inflammation during NAFLD. Findings rely on several pieces of evidence, e.g., in murine livers, increased FFA delivery induced oxidative metabolism, and amplified anaplerosis/cataplerosis with a proportional rise in oxidative stress and inflammation. The genetic knockdown of phosphoenolpyruvate carboxykinase 1 (Pck1) with loss of anaplerosis/cataplerosis prevented a fatty acid-induced rise in oxidative flux, oxidative stress, and inflammation. Flux was regulated by redox state, energy charge, and metabolite concentration, likely amplifying antioxidant pathways; the use of metformin to prevent increased oxidative metabolism was associated with both normalized hepatic anaplerosis/cataplerosis and reduced markers of inflammation. Additional findings suggest that histological grades in human NAFLD biopsies were proportional to oxidative flux. The evidence suggests that hepatic oxidative stress and inflammation are indeed associated with elevated oxidative metabolism during an obesogenic diet. An explanation might be the increased work through anabolic pathways. Obese individuals show that in the fatty liver, oxidative stress and inflammation parallel the elevated oxidative metabolism leading to increased anabolic pathways [118].

In addition, mitochondrial superoxide anion radicals/hydrogen peroxide [(*)O2(-)/H_2_O_2_] has deleterious effects on the development of metabolic diseases, including NAFLD [37]; breath testing using specific substrates points to mitochondrial abnormalities during liver steatosis [110,111,113,114,119,120,121,122]. Mitochondrial damage also includes: (a) the increased synthesis of mitochondrial free cholesterol due to (SREBP)-2-mediated upregulation of HMGCoA reductase, and apoptosis and the JNK-dependent proinflammatory pathways [89,123]; and (b) a decrease in nicotinamide adenine dinucleotide (NAD^+^/NADH) levels and involvement of the histone deacetylases, sirtuin-1 and -3, which modulate an adaptive response to increased hepatic levels of FFA [124].

A link exists between insulin resistance and mitochondrial abnormalities [101]. Impaired human plasma branched-chain amino acids (BCAA)-mediated upregulation of the TCA cycle can contribute to mitochondrial dysfunction in NAFLD [125]. There is a relationship between BCAA and insulin resistance, and the metabolic mitochondrial modulation is sensitive to overload from BCAA. These amino acids are essential to mediate efficient channeling of carbon substrates for oxidation through the mitochondrial TCA cycle. Mitochondrial genetics plays a role in NASH, and the mechanism implies the active modulation of oxidative stress and the efficiency of oxidative phosphorylation [126].

### 8.4. Nitrosative Stress and Cell Death

In NAFLD, the nitrosative stress (i.e., the overproduction of nitric oxide (NO), often accompanied by the simultaneous production of superoxide anions, which results in the formation of peroxynitrite and other reactive nitrogen species) contributes to cell damage. The locally produced nitric oxide derivatives can bind to certain protein thiols leading to enzyme inactivation and conformational changes in different membrane transporters [127]. NO modulates mitochondrial respiration and biogenesis [128]. Both ROS and NO can damage the mitochondrial function due to post-translational changes of the mitochondrial proteome. Studies on mitochondrial proteomics suggest that defects involve the assembly of multiprotein complexes and highly hydrophobic proteins of the inner mitochondrial membrane [129].

All the above-reported steps could lead to hepatocyte death [130] because disruption of intracellular homeostatic processes and of mitochondrial function activate both necroptotic events and apoptotic signaling [131]. Necroptosis occurs in NASH [132]. Apoptosis occurs with the release of proapoptotic proteins from mitochondrial intermembrane space and changes in mitochondrial cardiolipin and phosphatidylcholine redox state. Other events lead to an increased probability of mitochondrial permeability transition pore (MPTP) opening [133]. MPTP is a pore through the mitochondrial membranes consisting of the voltage-dependent anion channel (VDAC) in the outer mitochondrial membrane and the adenine nucleotide translocator (ANT) in the inner mitochondrial membrane [134]. In a study in the homogenate of cerebellar granule cell en route to apoptosis, alteration of the adenine nucleotide translocator occurs, resulting in MPTP opening [135]. More recently, an involvement of ATP synthase in the pore formation has been proposed [136,137,138,139].

Induction of MPTP will immediately open the inner mitochondrial membrane with the release of potentially toxic levels of ROS. If ROS levels are raised during a prolonged time interval, however, the prolonged opening of the MPTP will lead to depolarization of mitochondria, failure of oxidative phosphorylation, ATP depletion, and the release of proapoptotic factors and eventually rupture of the outer mitochondrial membrane [140]. Notably, ROS and lipid peroxidation increase in individuals with steatosis and NASH [101]

Release of cytochrome c and other proapoptotic factors into the cytosolic compartment, lysosomal damage, oxidative stress, and MPTP opening is likely to activate the NLR family pyrin domain-containing 3 (NLRP3) protein that functions within the NLRP3 inflammasome with the executioner caspase 3 interacting with pro-caspases 6, 7, and 2 [127,141].

The composition of the mitochondrial membrane, especially of the inner mitochondrial membrane, can also change with liver steatosis, with qualitative/quantitative transformation of cardiolipin, a phospholipid critical in many reactions and processes related to mitochondrial function and dynamics [107,142]

Lipid peroxidation and oxidative DNA damage proved to increase in NASH individuals, as shown by measuring the levels of the markers 8-hydroxydeoxyguanosine (8-OHdG) and HNE [143], and an increase in systemic inflammation was also found [116]. In the long term, liver steatosis can also induce endoplasmic reticulum stress, increased levels of Ca^2+^ in the mitochondrial matrix, apoptosis, and MPTP opening [144].

## 9. Therapy of NAFLD

To date, no ultimate therapy for NAFLD/NASH has been accepted by the Food and Drug Administration (FDA) or the European Medicines Agency (EMA). This limitation depends on complex pathogenic pathways involved, on the short duration of available trials, and on the potential additive (but still uninvestigated) effects of combined treatments. Much of the attention is currently focusing on NASH and liver fibrosis because both conditions are associated with a significant risk of progression to severe, end-stage liver disease. This is the current policy at EMA and FDA since monotherapy is associated with histological improvement of NASH in about 30–40% of the patients, as compared with placebo treatment. The complexity of the pathogenetic pathways involved in NAFLD/NASH accounts for the difficulty of identifying a specific therapeutic agent as monotherapy. Combination therapy, in this respect, is an emerging field of investigation, i.e., combining agents acting at a metabolic level with drugs acting on liver steatosis, or inflammation, or fibrosis, and therefore, targeting specific subgroups of patients. As of May 2021, interventional studies ongoing at www.clinicaltrial.gov (accessed on 19 May 2021) were less than 200, either as monotherapy or (few) combination therapies. Modification of lifestyles and general measures are the first step for treating NAFLD/NASH.

### 9.1. Modification of Lifestyles and General Measures

Modification of dietary habits and lifestyles is the first step for treating NAFLD/NASH. Especially in overweight/obese subjects, it is important to reach and maintain the ideal body weight [145]. Weight loss should be 5–7% in NAFLD and 7–10% in NASH in both overweight and obese patients [46]. This strategy might improve liver biochemical tests, liver histology, serum insulin levels, and quality of life [146,147,148,149,150,151]. With at least 10% weight reduction, liver fibrosis can improve in NASH. This goal, however, is difficult to maintain for a long time and to achieve in the majority of patients [149,152]. A healthy diet should be based on long-term caloric restriction rather than intermittent fasting to improve insulin sensitivity [153] and to prevent oxidative damage [154,155]. Subjects should avoid adding sugars, including fructose, in drinks and foods [156]. The Mediterranean diet might play a beneficial role [37,157]. If weight loss is insufficient and patients meet the inclusion criteria, bariatric surgery may be performed to reduce the prevalence of NASH [158,159]. However, a follow-up is required because of the potential worsening of fibrosis [160,161,162,163,164,165,166]. Physical exercise plays an important role in achieving weight loss or maintaining ideal weight. In the rodent model, endurance training mitigates the clinical/anatomical-related features induced by the Lieber-DeCarli diet, and this approach may reduce the risk of developing obesity and metabolic disorders [167]. It is recommended to take general measures in NAFLD patients. Patients should be assessed to reduce the risk factors for cardiovascular disease [64], to control diabetes mellitus, and to start a lipid-lowering therapy. Alcohol abstinence is important in NAFLD since alcohol consumption, even in small amounts, is associated with the progression of liver fibrosis [168]. In addition, intestinal bacteria contribute to the formation of endogenous ethanol, which has been shown to induce mitochondrial dysfunction in NAFLD [169]. Vaccination for hepatitis A virus and hepatitis B virus is recommended in patients without serologic evidence of immunity, while other vaccinations are like the rest of the population.

### 9.2. Drugs

**Figure 5:**
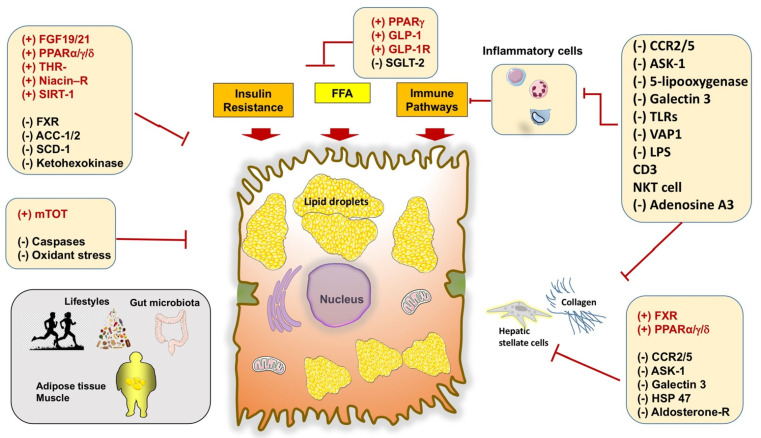
Potential therapeutic targets for NASH, as available from phase 2 and 3 clinical trials. Sites of action include liver pathways involved in lipid and glucose homeostasis, oxidative stress, mitochondrial function, inflammatory signals, intracellular targets related to stellate cell activation and fibrogenesis. Some targets (e.g., FXR agonists, C-C motif chemokine receptor [CCR] 2 and 5 (CCR2/5) antagonist) display more than one action site. Additional extrahepatic interventions appear in the left lower box. Symbols point to agonists (+) or antagonist (-) effect. Abbreviations: DGAT, diacylglycerol O-acyltransferase; SCD, steroyl CoA-desaturase; THR, thyroid hormone receptor; SIRT, sirtuin; GLP, glucagon-like peptide; SGLT, sodium-glucose cotransporter; VAP, vascular adhesion protein; LPS, lipopolysaccharide; PPARα/δ/γ, peroxisome proliferator-activated receptors PPARα, PPARδ and PPARγ [66].

Novel drugs and clinical trials are becoming available for NAFLD patients, but agents are still under experimental research. Potential targets include the dysfunctional pathways such as oxidative stress, apoptosis, glucose, and lipid metabolism, innate immunity, bile acid metabolism, nuclear receptors, liver fibrosis, i.e., fibrogenesis plus fibrinolysis, gut microbiota, and intestinal permeability [3,66,170,171] (Figure 5). A Cochrane review focused on 77 available trials on antioxidants, bile acids, and thiazolidinediones vs. no intervention and concluded that “Due to the very low-quality evidence, we are very uncertain about the effectiveness of pharmacological treatments for people with NAFLD including those with steatohepatitis. Further well-designed randomized clinical trials with sufficiently large sample sizes are necessary” [172].

Indeed, several limitations exist with therapy: (a) a single therapy leads benefits in no more than 40% of patients; (b) the trials conducted in NAFLD are too short to be recommended for life; and (c) combination therapies might increase the success rate of agents for NAFLD/NASH. Current and experimental therapies for NAFLD patients are depicted in Table 3.

## 10. Therapies Targeting Mitochondria in NAFLD

**Figure 6:**
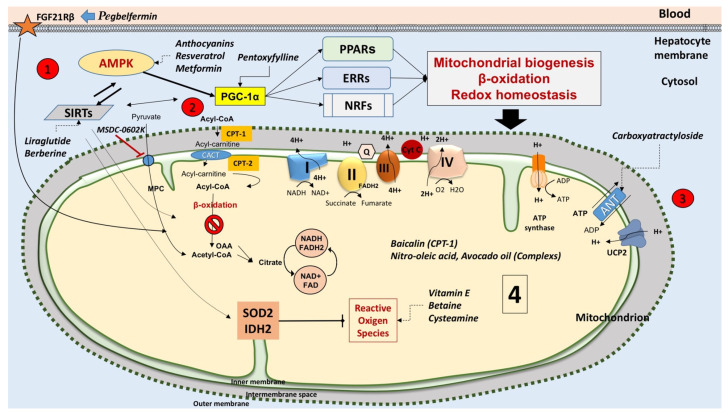
Mitochondria as targets in the NAFLD therapy. To develop a mitochondria-targeted therapy in NAFLD, a variety of drugs have been tested both in cellular/animal models and in very early clinical studies. The possible mitochondrial targets include: (1) nuclear receptors and compounds involved in different signaling pathways; (2) mitochondrial transporters; (3) enzymes playing a major role in mitochondrial metabolism; and (4) biomolecules involved in pathways controlling reactive oxygen species (ROS) and oxidative stress. Red lines indicate inhibition. Abbreviations: AMPK, AMP-activated protein kinase; FGF21Rβ, fibroblast growth factor 21 receptor β; SIRTs, sirtuins; PGC-1α, peroxisome proliferator-activated receptor coactivator 1α; PPARs, peroxisome proliferator-activated receptors; ERRs, estrogen-related receptors; NRFs, nuclear respiratory factors. ANT, adenine nucleotide translocator; UCP, uncoupling proteins; Cyt. C, cytochrome c; CPT-1, carnitine palmitoyl-transferase 1; CPT-2, carnitine palmitoyl-transferase 2; MPC, mitochondrial pyruvate carrier; SOD2, superoxide dismutase 2; IDH2, isocitrate dehydrogenase 2 [261].

Few therapeutic approaches target different pathways in NAFLD and could also be effective on dysfunctional mitochondria (Table 4 and Figure 6). Antioxidants targeting mitochondrial (*)O_2_(-)/H_2_O_2_, for example, represent one attractive strategy to counteract liver inflammation in NASH [257,258]. Definitive approaches, however, await further evidence.

### 10.1. Physical Exercise

Physical activity may improve NAFLD, and the mechanisms involve the modulation of function and structure of mitochondria [296]. Physical exercise induces mitochondrial biogenesis in the striated muscle and also in the liver [297]. A single acute bout of physical exercise has effects on hepatic metabolic and redox state, but it remains uncertain about the effects on mitochondrial membrane permeability to protons, state 4 respiration, increased state 3 respiration, and stress, response to mitochondrial permeability transition [298,299]. By contrast, long-term physical activity as endurance training (or voluntary running) might ameliorate markers of liver mitochondrial integrity and function and appear to facilitate hepatic features, which are typical of a phenotype that is more resistant to stress [299].

Animal models of NAFLD point to a link between physical exercise and mitochondrial function, as seen in Otsuka Long-Evans Tokushima Fatty (OLETF) rats that develop type 2 diabetes and obesity as stigmata of the metabolic syndrome. However, following 16 or 36 weeks of daily voluntary wheel running, rats display increased hepatic mitochondrial FFA oxidation, as well as enhanced oxidative enzyme function and protein content. In addition, levels of proteins related to hepatic de novo lipogenesis are suppressed [300,301]. Changes develop with several markers of mitochondrial oxidative phosphorylation apparatus and include increased palmitate oxidation, increased activities of β-hydroxyacyl-CoA dehydrogenase and carnitine, citrate synthase, palmitoyl-CoA transferase 1, cytochrome c, and ETC complex IV. Physical exercise also leads to an increase in the phosphorylated form of acetyl-CoA carboxylase (ACC) and a decrease in activities of ACC, FA synthase, and stearoyl-CoA desaturase (SCD), i.e., markers of inhibition of de novo hepatic lipogenesis [302]. In addition, the beneficial effects of exercise include the increase in the hepatic mitochondrial oxidative capacity associated with increased FFA oxidation and decreased FA-derived ceramide and diacylglycerol synthesis due to decreased insulin resistance [303,304].

**Figure 7:**
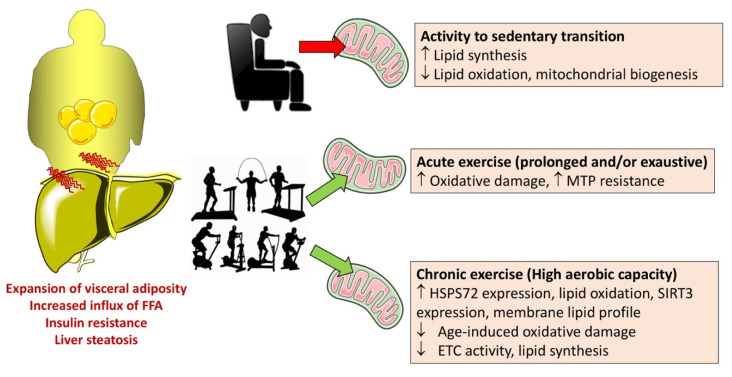
Liver tissue and mitochondrial adaptations by acute or chronic physical exercise, high aerobic capacity, and sedentary behaviors. Abbreviations: ECT, electron transport chain; ESP, heat shock proteins; MTP, mitochondrial permeability transition pore; SIRT, sirtuin. Arrows indicate an increase (↑) or a decrease (↓). Adapted from [299]. Cartoons obtained from http://www.riskmanagement365.com/wp-content/uploads/2013/03/physical-exercise.jpg and http://johannesbrug.blogspot.com/2015/10/determinants-of-engaging-in-sedentary.html (accessed on 19 May 2021) [305].

Comparison between effects of physical activity and those of sedentary behavior is shown in Figure 7.

### 10.2. Antidiabetic Drugs

Peroxisome proliferator-activated receptors (PPARs) belong to the superfamily of nuclear receptors. PPARs bind several FFA and FFA derivatives and regulate intracellular metabolic processes at a transcriptional level [306]. PPAR-α, PPAR-δ (also named PPAR-β), and PPAR-γ are the three subtypes of PPARs that display different functions in ligand selectivity and tissue distribution. PPAR-α is expressed mainly in the liver, adipose tissue, heart, skeletal muscle, and kidney and regulates lipid transport, gluconeogenesis, and the hormone called fibroblast growth factor (FGF)-21. Notably, the activation of PPAR-α shifts hepatic metabolism toward FFA transport and β-oxidation [171]. This activation improves plasma lipid profile, i.e., decreases TG and increases high-density lipoprotein (HDL) cholesterol [307]. PPAR-α also displays anti-inflammatory effects in the liver via upregulation of anti-inflammatory genes, such as *Il-1ra* and *IkB*α, a cytoplasmic inhibitor of NF-kB, and this effect points to the cooperation between PPAR-α-dependent transactivation and transrepression to turn on anti-inflammatory pathways [307]. PPAR-α deletion in the animal model is associated with worsening of liver steatosis. However, fibrates (typical PPAR-α agonists), while lowering serum TG concentrations, show no effect on NAFLD [308].

PPAR-δ receptors are expressed largely in the liver, skeletal muscle, and macrophages, as well as ameliorate insulin sensitivity and decrease hepatic glucose production [309]. In addition, PPAR-δ activation increases FFA oxidation and decreases macrophage and Kupffer cell activation [310]. PPAR-δ has anti-inflammatory activities in the liver, acting on macrophages and Kupffer cells [310]. Despite the activation of PPAR-δ decreases liver steatosis, there are concerns about its safety [311].

Elafibrinor is effective as a PPARα/δ agonist, and increases FFA β-oxidation (PPARα activity), as well as also improves insulin resistance and inflammation (see above) [309,310]. The potential effect on liver mitochondria is under investigation.

PPAR-γ is expressed mainly in the adipose tissue and regulates lipogenesis, glucose metabolism, and differentiation of the adipocytes. The class of thiazolidinediones (TZDs) are PPARγ agonists, acting as insulin sensitizers and antidiabetic drugs (Table 3). TZDs have an effect on NAFLD in patients [173,180,312] since pioglitazone [173,178,179,180,181] and rosiglitazone [185,186,187] improve NASH. A mitochondrial target of thiazolidinediones could be mTOT, a mitochondrial membrane complex involved in pyruvate transport. TZDs, therefore, could modulate the entry of pyruvate into the TCA cycle [313]. Indeed, liver steatosis is associated with increased activity of the TCA cycle and decreased availability of acetyl-CoA [117]. In the mouse model of NASH, pioglitazone can partly ameliorate this situation while decreasing the hepatic TCA cycle flux [314]. The mechanism might involve inhibition of mitochondrial pyruvate fluxes [315,316]. In addition, MSDC-0602K is a novel PPARγ agonist, and as an insulin sensitizer, targets the mitochondrial pyruvate carrier while minimizing direct binding to the transcriptional factor [189].

Novel antidiabetic drugs such as liraglutide (glucagon-like peptide 1 analog and GLP-1 receptor agonist) [198] and sitagliptin (dipeptidyl peptidase-4 inhibitor) [317,318] might be effective in treating NAFLD, but drugs require more clinical evidence.

Mitochondrial effects derive from pioglitazone in nephrectomized rats. The drug prevents the leakage of cytochrome c from the mitochondria, stabilizes the mitochondrial transmembrane potential, inhibits ROS generation, and activates the electron transport chain complexes I and III. In the model, pioglitazone has reno-protective effects through modulating mitochondrial electron transport chain and mitochondrial dynamics while protecting against fibrosis [264]. Mitochondrial dysfunction in NAFLD might represent another model to test the efficacy of TZDs and the role of novel drugs such as selective modulators of PPARα (pemafibrate and K-877), and PPARγ (INT-131), PPARδ (HPP-593), and PPARα/γ (DSP-8658) agonists [171].

Metformin (dimethylbiguanide) is another interesting drug. It improves hepatic and peripheral tissue sensitivity to insulin. In cultured HepG2 cells loaded with oleic acid to induce steatosis, metformin decreases steatosis and improves hepatocyte function. Several mechanisms are involved, which include decreased oxidative stress injury, regulation of protein expression related to the mitochondrial apoptosis pathway, and the inhibition of cell apoptosis [319]. Notably, metformin activates AMPK to stimulate mitochondrial biogenesis and FFA β-oxidation [263].

Liraglutide is an acylated glucagon-like peptide-1 (GLP-1) agonist. In cultured HepG2 cells, it improves NASH. The mechanism relies on the inhibition of the nucleotide-binding oligomerization domain, leucine-rich repeat-containing receptor-containing pyrin domain 3 (NLRP3) inflammasome, and pyroptosis activation via mitophagy [320]. In HFD-fed mice, liraglutide ameliorates NAFLD by enhancing mitochondrial architecture, attenuating ROS production, and promoting autophagy through the SIRT1/SIRT3 pathway [262].

### 10.3. Bile Acids (BA)

BA are soluble amphiphilic molecules and major lipid components of bile with phospholipids and cholesterol. The liver is the site where primary BA, i.e., cholic acid (CA) and chenodeoxycholic acid (CDCA), is synthetized from cholesterol and conjugated to the amino acids glycine or taurine to increase its solubility in bile. BA is then actively secreted into bile, concentrated in the gallbladder during fasting, and released into the duodenum after dietary fat-induced neuro-hormonal stimulation of the gallbladder. Flowing through the intestine, primary BA is bio-transformed to secondary BA, i.e., deoxycholic acid (DCA) and litocholic acid (LCA), and tertiary BA, i.e., ursodeoxycholic acid (UDCA), by the gut microbiota. Both primary and secondary/tertiary BA are reabsorbed in the ileum and the colon, respectively, and then recirculated to the liver via the portal tract, with minimal fecal loss. Besides their digestive function for fat micellization, more lipophilic BA also plays a role as signaling molecules in modulating epithelial cell proliferation, gene expression, and lipid and glucose metabolism. This function occurs by activation of the nuclear farnesoid X receptor (FXR) and membrane-associated G-protein-coupled bile acid receptor-1 (GPBAR-1) in the liver, ileum, muscle, and brown adipose tissue [26,28]. In the liver, the BA-FXR interaction inhibits BA synthesis and acts transcriptionally to decrease hepatic lipogenesis and steatosis [321]. In addition, hepatic gluconeogenesis and peripheral insulin resistance are also decreased [322].

OCA, the lipophilic synthetic variant of CDCA, acts as an FXR agonist and decreases hepatic lipogenesis because of the downregulation of the transcription factor SREBP1c and upregulation of SIRT1 [323,324]. In addition, FXR activation leads to intestinal production of the enterokine fibroblast growth factor 19 (FGF19) that binds the hepatic FGF receptor (FGFR)4 and promotes mitochondrial FFA β-oxidation and hepatic glycogen synthesis [26,325].

UDCA, the epimer of CDCA, has hepatoprotective effects in patients with several chronic liver diseases [326]. UDCA has some beneficial effects on liver enzymes and biopsy-proven NASH in an open-label pilot study [327]. In a subsequent randomized trial, 166 patients with liver biopsy-proven NASH are randomized to receive oral UDCA at 13–15 mg/kg/daily or placebo for 2 years. Finally, 126 patients complete the study, with 107 liver biopsies available. The results show that UDCA is safe but has no advantage over placebo concerning serum liver biochemistry, degree of steatosis, necroinflammation, or fibrosis. UDCA has hydrophilic properties but a low affinity for FXR or might even antagonize FXR activity [328]. No information is available about analogs of UDCA, i.e., tauroursodeoxycholic acid (TUDCA) or nor-UDCA. Notably, lipophilic BA, i.e., DCA, CDCA, and LCA, inhibits the mitochondrial electron transport chain. At high BA concentrations (100 μmol/L), the effects on the inner mitochondrial membrane of intact mitochondria are not specific. Low BA concentrations (10 μmol/L), however, have specific effects, i.e., impairment of complex I and complex III, on broken mitochondria or on intact mitochondria [329]. Mitochondrial antioxidative capacity decreases during chronic cholestatic liver disease when excess retention of BA occurs [330]. Most, but not all, BA can alter mitochondrial bioenergetics with concentration-dependent effects [331]. UDCA, for example, has antioxidant and anti-inflammatory properties and prevents mitochondrial dysfunction during the progression of obesity-associated complications. In the isolated rat liver, it is investigated about the protective effects of hydrophilic UDCA and TUDCA, as well as the toxicity of lipophilic CDCA and LCA on the function of the electron transport chain in mitochondria [332]. The results show that CDCA and LCA reduce state 3 oxidation rates and respiratory control ratios of L-glutamate, succinate, and duroquinol, at a concentration of 30 μmol/L, without affecting ADP/O ratios (i.e., ratio of added ADP and oxygen consumed) of these substrates and oxidative metabolism of ascorbate. UDCA, up to 100 μmol/L, does not interfere with mitochondrial oxidative metabolism, while at 300 μmol/L, it has an effect similar to CDCA and LCA. When the concentration is as high as 300 μmol/L, TUDCA has no obvious inhibitory effect. The toxic effects of CDCA and LCA on mitochondrial oxidative metabolism are partially reversed with UDCA at 30 μmol/L or 100 μmol/L, whereas UDCA at 300 μmol/L plus CDCA or LCA produces greater toxicity compared with individual BA. TUDCA does not reduce the toxic effects of CDCA or LCA on mitochondrial metabolism. Together, these results indicate that BA has a distinct effect on mitochondrial oxidative metabolism. When the concentration is as high as 100 μmol/L, UDCA decreases the toxicity of lipophilic BA on the function of the electron transport chain. However, at higher concentrations, UDCA increases BA-induced mitochondrial toxicity. Likely, the incorporation of BA into mitochondrial membranes is decreased. The protective effects of UDCA might go beyond the simple action on mitochondria and involve several other mechanisms of metabolic damage, mimicking a multi-target therapeutic agent. UDCA modulates glucose and lipid biosynthesis, inflammatory response, angiogenesis, and macrophage differentiation in ob./ob mice. UDCA significantly reduces lipid droplet formation, as well as FFA and TG concentrations, improves mitochondrial function, and enhances white adipose tissue browning. In addition, UDCA increases hepatic energy expenditure, mitochondria biogenesis, and incorporation of BA metabolism through *Abca1* and *Abcg1* mRNA, and BSEP, FGFR4, and TGR5 proteins, and downregulates NF-kB and STAT3 phosphorylation through negative regulation of the expression of *SOCS1* and *SOCS3* signaling. These changes occur together with decreased angiogenesis through downregulation of *VEGF*, *VCAM*, and *TGF-betaRII* expression. UDCA also reduces whole-body adiposity while decreasing expression of macrophage CD11b, CD163, and CD206 in the adipose tissue, as well as levels of lipogenic capacity markers such as lipofuscin, SREBP-1, and CD36. UDCA also upregulates adipose browning in association with the upregulation of SIRT-1-PGC1-alpha signaling in epididymis adipose tissue (EWAT) [265].

Aramchol (arachidyl-amido cholanoic) has some beneficial effects on liver steatosis in humans but does not improve liver enzymes, glucose metabolism, and insulin sensitivity [216,217]. In the animal model, aramchol treatment improves steatohepatitis and fibrosis by decreasing stearoyl-coenzyme A desaturase 1 (SCD1) and increasing the flux through the trans-sulphuration pathway maintaining cellular redox homeostasis [216]. SCD1 deficiency in mice reduces lipid synthesis and increases mitochondrial FFA β-oxidation in the mitochondria and insulin sensitivity in various tissues, including the liver. In this context, SCD1 deficiency has been demonstrated to prevent liver steatosis in several mouse models of NAFLD, e.g., mice fed the high-carbohydrate and high-fat diet.

### 10.4. Antioxidant Agents

There is a lack of solid evidence concerning the effect of antioxidants on NAFLD because of several confounding factors [333]. The correction of the oxidative stress in NAFLD does not seem to interfere with the general redox homeostasis. In addition, it is important to assess the cell specificity of antioxidative therapy and ultimately identify reliable biomarkers of liver damage [334]. The origin of oxidative stress in NAFLD is a complex and multifactorial process, and antioxidant agents might act by non-specific mechanisms or have poor efficacy, while many available antioxidant agents show poor cell membrane permeability and selectivity.

Vitamin E (α-Tocopherol) [173] at a high dose has some effects on biopsy-proven NASH and fibrosis stage ≥2, but not diabetes mellitus, and improved liver steatosis and fibrosis [64] in patients (see Table 3).

Tempol, a nitroxide (4-hydroxy-2,2,6,6-tetramethylpiperidine-*N*-oxyl), undergoes a one-electron reduction reaction to form hydroxylamines or two-electron reduction reactions to form oxammonium cations. Because of this redox effect and other actions, Tempol might be beneficial in NASH. The gut microbiota is part of the complex gut-liver axis [28], and tempol is able to modulate the composition and metabolism of the gut microbiota under pro-steatotic conditions. This effect improved liver histology, as shown by the marked reduction in lipid droplets. Tempol is also effective in decreasing liver weight and liver/body mass ratios by interfering with the intestine-specific disruption of FXR in mice fed a steatogenic diet [335]. The intestinal FXR influences the ceramide/SREBP1C/CIDE-A (Cell death activator CIDE-A) pathway, and administration of ceramide attenuates the effects of the steatogenic diet on steatohepatitis. The inhibition of intestinal FXR is therefore crucial for gut microbiome-mediated progression of NAFLD. The inhibition of intestinal FXR signaling also can improve the mitochondrial function, likely repressing the synthesis and serum levels of ceramide and then decreasing hepatic steatosis [266].

Resveratrol is the polyphenol extract obtained from red grapes and berries. The agent has a protective effect on mitochondria [267], decreases the steatosis induced by a high-fat diet (HFD), and acts on the pathways that are involved in the regulation of AMPK and SIRT1 [268,269,270]. There are no sound data about the clinical benefit of resveratrol in NAFLD [336], which produces attenuation of liver fibrosis via the AKT and NF-κB pathway without affecting fat accumulation in the liver.

Other antioxidant agents are designed to target mitochondria, but the results are weak. Some molecules transport and concentrate antioxidant molecules within mitochondria [271,272,273], such as mitoquinone (Mito-Q) and mitovitamin E (MitoVit-E), which display a covalently attached lipophilic triphenylphosphonium (TPP) cationic moiety. Mito-Q is able to improve the metabolic syndrome in rats fed the high-fat diet for eight weeks [337]. In liver mitochondria, the effect is associated with increased expression of cardiolipin synthase and cardiolipin levels [338]. In addition, Mito-Q prevents metabolic abnormalities such as hypertriglyceridemia, hypercholesterolemia, hyperglycemia, hepatic steatosis, and mtDNA oxidative damage in experimental models of the metabolic syndrome and atherosclerosis [339]. Low doses of Mito-Q and MitoVit-E protect cells against peroxide-induced oxidative damage and apoptosis. This effect is in contrast to what occurs with low doses of untargeted antioxidants, e.g., Vit-E and ubiquinone. The protective effects of Mito-Q and MitoVit-E are likely mediated through the inhibition of cytochrome c release and caspase-3 activation. Mito-Q and MitoVit-E reduce ROS-induced transferrin receptor-mediated iron uptake in mitochondria, lipid peroxidation, lipid peroxide-induced inactivation of complex I, and aconitase [340]. A phase II study in patients with chronic hepatitis C shows that Mito-Q decreases circulating aminotransferase levels. The effect points to reduced hepatic inflammation and necrosis [341].

Pentoxyfylline improves histological features of NASH in a randomized and placebo-controlled trial [283,284]. Interestingly, pentoxyfylline might upregulate mitochondrial biogenesis and FFA β-oxidation via increased expression of PGC1α and its downstream or parallel PPARα [282].

Medicinal plants may be used as dietary supplements [342]. Silymarin is extracted from milk thistle (*Silybum marianum*), and silybin is its major active compound that has some hepatoprotective effects [343]. Studies suggest that silybin might improve insulin resistance, liver injury, and hepatic enzymes in NAFLD patients [344,345]. Moreover, the silybin-phospholipid complex enriched with vitamin E improves liver steatosis in NAFLD patients [346]. In parallel, silybin significantly lowers fat infiltration in the liver of rats fed the high-fat diet. The mechanism implies the modulation of thioredoxin changes and the synthesis of nitric oxide (NO) derivatives. Lipid peroxidation is also significantly lowered. In mitochondria, silybin mitigates changes in mitochondrial respiratory complexes and has a major protective effect on complex II subunit CII-30 [274]. Silybin is also protective on rat hepatoma FaO cells that are challenged by FFA to develop a progressive model of liver steatosis [275]. Silybin reduces excessive TG accumulation and changes the expression of transcription factors such as PPARs, enzymes involved in mitochondrial, endoplasmic reticulum, and peroxisomal oxidation of FFA. Silybin also rescues the FFA-induced mitochondrial dysfunction, i.e., size and function, and the apoptotic signals and oxidative stress, in a cellular model resembling steatohepatitis [108].

Corilagin, a polyphenol tannic acid compound retrieved in many ethnopharmacological plants, shows antioxidant properties [276]. Corilagin reduces lipid deposition of diet-induced NAFLD in the animal model with a decrease in oxidative stress and restoration of autophagic flux. Mitochondrial function is improved through decreased mtDNA oxidative damage and increased mitochondrial biogenesis-related transcription factors expression, mitochondrial DNA content, as well as oxygen consumption rate.

Anthocyanins are plant flavonoids contained in the berries of bilberry and black currant. These compounds activate AMPK and its downstream PGC-1α. Beneficial effects include restoring mitochondrial content, biogenesis, OXPHOS, and FFA β-oxidation in mice because these pathways govern oxidative stress, steatosis, inflammation, and fibrosis [277,278].

Dihydromyricetin is a type of flavonoid found in several natural plants, including *Ampelopsis species japonica*, *megalophylla*, and *grossedentata*, *Cercidiphyllum japonicum*, *Hovenia dulcis*, *Rhododendron cinnabarinum*, some Pinus species, some Cedrus species, and *Salix sachalinensis*. In mice fed with the high-fat diet and in hepatocytes treated with palmitic acid, dihydromyricetin improves NAFLD. The mechanism involving SIRT3 improves mitochondrial respiratory capacity and redox homeostasis in the hepatocytes and decreases hepatic lipid accumulation and oxidative stress [279].

Berberine (isoquinoline alkaloid) increases mitochondrial SIRT3 activity and improves OXPHOS in the liver of rats fed with a high-fat diet [255].

As an antioxidant strategy, impediment of mitochondrial ROS production through uncoupling might be a valid alternative to the removal of ROS by using antioxidants. The artificial uncoupler 2,4-dinitrophenol has toxic effects [347]. Further studies need to assess the ultimate role of this therapeutic strategy, especially in NAFLD [348]. Controlled-release mitochondrial protonophore (CRMP) is a controlled-release oral formulation of DNP that produces mild hepatic mitochondrial uncoupling. In rat models, CRMP reduces hypertriglyceridemia, insulin resistance, hepatic steatosis, and diabetes. CRMP also normalizes plasma transaminase concentrations, ameliorates liver fibrosis, and improves hepatic protein synthetic function in a methionine/choline-deficient rat model of NASH. There was no systemic toxicity [349].

The antioxidant hydroxytyrosol (HT) shows some beneficial effects also on mitochondrial function in mice fed with the high-fat diet and treated with n-3 LCPUFA eicosapentaenoic acid [249].

Cysteamine is an aminothiol and acts as a scavenger of ROS. This step parallels the enrichment of glutathione stores, with a potential benefit for NAFLD. Hepatic enzymes are improved in children with biopsy-proven NAFLD, but liver histology or NASH does not improve [280,281].

Avocado oil represents a rich source of C18:1 bioactive sterols and antioxidants. In mitochondria, it might decrease the unsaturation of acyl chains of membrane lipids and/or improve the electron transport chain functionality with decreased ROS generation. In the rat model of streptozocin-induced diabetes also manifesting NAFLD, avocado oil decreased mitochondrial oxidative stress and lipid peroxidation with enhanced complex I activity and attenuation of ROS production [286]. The same authors fed rats with a diet with high fat and fructose for 4 months to induce several alterations in the liver (inflammation, ballooning, necrosis), serum (increased expression of cytokines TNF-α and IL-6), and mitochondria (ROS production and lipid peroxidation). Avocado oil administration counteracted these abnormalities suggesting that in NAFLD, avocado oil can decrease inflammation and improve mitochondrial dynamics. According to such evidence, avocado oil may be a nutritional approach to complement the pharmacological treatment of NAFLD [287]. The translational value of such observations requires caution since others recapitulated a potential detrimental effect of avocado oil uncoupler on mitochondria of steatotic-diabetic rats [285]. In light of these aspects, the ultimate efficacy of avocado oil in humans is controversial.

### 10.5. Mitotherapy

Mitochondria are mainly responsible for energy supply in mammalian cells, and over 100 human diseases are attributed to mitochondrial dysfunction. The concept of mitochondrial therapy (mitotherapy) defines the transfer of functional exogenous mitochondria into mitochondria-defective cells. This sequence is associated with recovery of the cell viability and possibly, prevention of the disease progress [350]. Exogenous intravenous injection of functional mitochondria from hepatoma cells might successfully improve the phenotype of high-fat diet-induced liver steatosis by decreasing lipid content and improving cellular redox balance. Exogenous mitochondria tagged with green-fluorescence protein (GFP) are retrieved in mouse liver, lungs, brain, muscle, and kidneys [288,289]. This experimental protocol should decrease lipid deposits, prevent cell injury, improve energy production, and restore hepatocyte function. More studies should clarify how mitochondria enter different cells restoring the cellular metabolic activity [290]. Aspects related to the nature of the administrated mitochondria, distinct metabolic and proteomic differences in mitochondrial isolated from normal, non-tumor-derived hepatocytes deserve further studies.

### 10.6. Novel Agents

Much attention is being given to novel agents active on mitochondrial function. More evidence is required in this respect.

Aramchol could improve NAFLD/NASH by acting on mitochondrial function. In mice, SCD1 deficiency results in reduced lipid synthesis and increased mitochondrial FFA β-oxidation and insulin sensitivity in various tissues, including the liver [351,352]. In a mouse model of NASH, by feeding the methionine- and choline-deficient (MCD) diet for 4 weeks, administration of aramchol at 5 mg/kg/day for the last 2 weeks improves steatohepatitis and fibrosis by decreasing SCD1. Aramchol increases the flux through the trans-sulphuration pathway, leading to a rise in glutathione (GSH) and the GSH/oxidized GSH ratio, the main cellular antioxidant that maintains intracellular redox status [216].

Baicalin is the flavonoid component of the herbal medicine, *Scutellaria baicalensis*. In in vitro cell culture of hepatocytes and mouse model, baicalin directly activates hepatic CPT1 and accelerates the lipid influx into mitochondria for FFA β-oxidation. Indeed, chronic treatment of baicalin ameliorates diet-induced obesity and hepatic steatosis with the improvement of other metabolic disorders. The finding that baicalin functions as an allosteric CPT1 activator opens a new opportunity for pharmacological treatment of diet-induced obesity and associated sequelae [291].

Nitro-oleic acid treatment improves the function of hepatic mitochondrial complexes I, IV, and V and decreases oxidative stress, with protection from diet-induced hepatic steatosis in mice [292].

Systemic administration of a low dose of carboxyatractyloside, a specific inhibitor of ANT, might protect against fatty liver in mice [293].

Genistein, an isoflavone reported to prevent apoptosis in cerebellar granule cells [353], does not inhibit hepatic steatosis but attenuates steatohepatitis induced in the methionine-choline-deficient (MCD) diet-fed mice. The mechanism includes AMPK inactivation and inhibition of inflammation [294].

GS-0976 is a potent acetyl-CoA carboxylase (ACC) inhibitor and is also effective on mitochondrial ACC2 (see above for detailed discussion). If ACC is inhibited, mitochondrial FFA β-oxidation is increased, and this effect might reduce hepatic steatosis and fibrosis [200].

## 11. Combination Therapy

**Figure 8:**
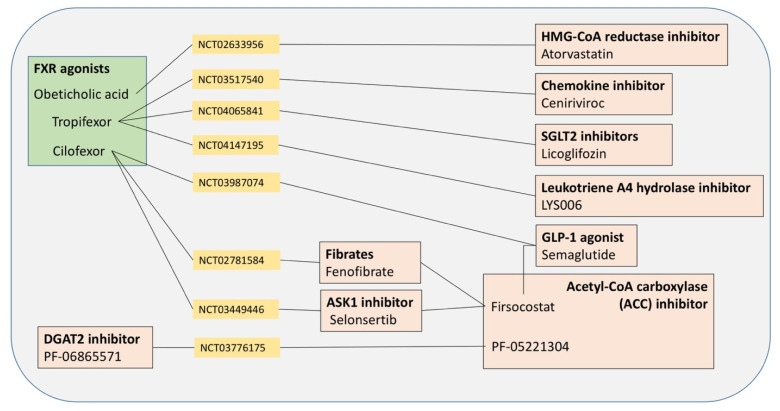
Trials of combination therapies in progress for the treatment of nonalcoholic steatohepatitis (NASH) [204]. The number of trial (NCT) is derived from www.clinicaltrial.gov (accessed on 19 May 2021).

As assessed by histological improvement of liver fibrosis in NASH patients, therapies confirm that only about 30% of patients improve the histological picture, as compared with the placebo group [204]. The complexity of NAFLD and NASH is partly accounting for this poor therapeutic outcome. Combination therapy might play a better role than monotherapy in this respect, e.g., combining a drug with a metabolic mechanism of action with a drug with an anti-inflammatory or an antifibrotic mechanism of action. The rationale for combining at least two drugs includes enhancing efficacy (because of increased response rate, increasing response rate, and reduced loss of effects due to prolonged treatment) and improving tolerability across the key pathogenetic sequence of steatosis, inflammation, and fibrotic changes [204]. Chronology of treatment can be different, depending on individual circumstances and protocol, i.e., overlapping treatment, outlasting treatment, and additional treatment [204]. Drug classes amenable to combination therapy include FXR agonists, PPAR agonists, metabolic enzyme inhibitors, thyroid hormone receptor beta-agonists, mitochondria pyruvate carrier inhibitors, FGF21 agonists, GLP-1 agonists, SGLT2 inhibitors, and chemokine inhibitors. Current studies on association therapies in patients with NAFLD/NASH are depicted in Figure 8. Depending on the association, studies have reported that there are decreased liver fat and serum liver enzymes (NCT02781584) and improved liver fibrosis (NCT03449446). Several trials, however, are currently in progress, and results are awaited. The idea of combining antidiabetic drugs with specific anti-NASH drugs could provide an additional advantage in terms of metabolic and liver (fibrosis-inflammation) conditions. Few trials are in progress in this area (NCT04065841), (NCT03987074). Minimizing the side effects is another example of using combination therapy, as shown in the (NCT02633956) trial, where the addition of atorvastatin counteracted the increase in LDL-cholesterol by OCA [354]. In another trial, the addition of fenofibrate before firsocostat in NASH fibrosis patients prevents hypertriglyceridemia and improves hepatic fat and liver biochemistry (NCT02781584) [295].

The approach of combining drugs may also be successful in ameliorating mitochondrial function, but further studies are awaited in this field.

## 12. Conclusions

NAFLD has become the most common chronic liver disease worldwide and represents the liver manifestation of metabolic syndrome. As NAFLD prevalence will increase, the prevalence of NASH, liver cirrhosis, and HCC will also inevitably increase. NAFLD is also associated with extrahepatic manifestations of cardiovascular disease. Due to the overall burden of the disease, both prevention and treatment of NAFLD at any possible stage gain paramount importance. Despite several new drugs and molecular targets are promising, many clinical trials conclude that the optimal pharmacological approach still to come due to the complex pathogenesis of NAFLD and NASH. One logical approach would be to target some subcellular organelles involved in the pathogenic process while searching for genetic risk variants and reducing metabolic and environmental stressors. Novel therapies may act both on mitochondrial function and energy supply other than on intracellular regulators of lipid metabolism. Adequate duration and power are needed to evaluate the long-term efficacy and safety of each potential therapeutic option. The beneficial effects of combination therapies are under close scrutiny and await convincing results.

## Figures and Tables

**Table 1:** The spectrum of nonalcoholic fatty liver disease (NAFLD).

Condition	Features
Nonalcoholic fatty liver (NAFL)	Simple steatosis No evidence of inflammation, necrosis, and fibrosis
Nonalcoholic steatohepatitis (NASH)	Steatosis associated with pericellular fibrosis, lobular inflammation, and apoptosis. Histological findings are indistinguishable from alcoholic steatohepatitis [57]. NASH affects 3% to 6% of the U.S. population with a risk of progression to cirrhosis in about 20% of the cases [58,59,60,61] Possible progression to hepatocellular carcinoma (HCC) [62]
Cryptogenic cirrhosis	Late stage of progressive hepatic fibrosis and steatotic chronic liver disease Distortion of the architecture of the liver and growth of regenerative nodules May progress to HCC
Hepatocellular carcinoma (HCC)	Primary tumor of the liver that usually develops in the setting of chronic liver disease

**Table 2:** Major events in the liver contributing to nonalcoholic fatty liver disease (NAFLD).

Outcome	Causes
Increased influx of the circulating free fatty acids (FFA)	Obesity
	Overfeeding (also saturated fatty acids)
	Rapid weight loss
	Total parenteral nutrition (transformation of carbohydrates/proteins to TG)
Decreased mitochondrial β-oxidation of FFA	Vitamin B5 (pantothenic acid) deficiency
	Excessive alcohol consumption
	Drugs: valproic acid or chronic aspirin → coenzyme A deficiency
	Decreased activity acyl-CoA:diacylglycerol acyltransferase 1 (DGAT1)
	Possible decreased activity of miRNAs
	Increased accumulation of ceramides
Decreased secretion/export of very low-density lipoproteins (VLDL)	Abetalipoproteinemia, protein malnutrition, or choline deficiency
	Defective secretion of postprandial Apo B
	Drugs: amiodarone and tetracycline → defective lipidation of Apo B (inhibition of microsomal triglyceride transfer protein (MTP))
	Ongoing NAFLD/NASH

**Table 3:** Ongoing clinical studies in NAFLD patients. Current and experimental agents are listed. NCT refers to ClinicalTrials.gov identifier number, as available at https://clinicaltrials.gov/ct2/home, accessed on 19 May 2021).

Class (Type of Compounds)	Observed Clinical Effects
Vitamin (Vitamin E)	- As an antioxidant agent [173], vitamin E could be used in patients with biopsy-proven NASH and fibrosis stage ≥2 but without diabetes mellitus. High doses of 800 IU/day improved steatosis and fibrosis [64].
Anti-apoptotic agents (Emricasan)	- Emricasan, a pancaspase inhibitor, inhibits liver injury, inflammation, and fibrosis [174,175]. More evidence required.
Insulin sensitizer (Metformin)	- Metformin has been suggested as the initial treatment of NAFLD patients with diabetes mellitus. However, no improvement in liver histology has been observed [64,176,177]
PPARγ-agonists (Thiazolidinediones: pioglitazone, rosiglitazone, MSDC-0602K)	- Pioglitazone is ineffective at the dose of 30 mg (PIVENS trial, NCT00063622). The dose of 45 mg improved liver fibrosis, inflammation, and steatosis [173,178,179,180,181,182]. - A metanalysis confirmed the effect of pioglitazone in NASH [183], but with increased risk of weight gain, heart failure, osteopenia, and fractures [184]. - According to the European Association for the Study of the Liver (EASL) and the American Association for the Study of Liver Diseases (AASLD), pioglitazone should be used in subjects with and without type 2 diabetes with biopsy-demonstrated NASH [56,64]. Pioglitazone, in fact, improved liver histology in these patients [178]. However, there is no indication to treat NAFLD without biopsy-based proof of NASH [56,64]. - Rosiglitazone has stronger PPARγ agonism than pioglitazone, with effects in NASH [185,186,187]. - In clinical practice, it is not advisable to use pioglitazone and rosiglitazone in NASH [188]. - MSDC-0602K might target the mitochondrial pyruvate carrier while minimizing direct binding to the transcriptional factor (EMMINENCE trial—NCT02784444) [189].
Dual PPAR activators (Elafibranor, Saroglitazar)	- Elafibranor (GFT505), an α/δ agonist, exerts antidiabetic effects in db/db mice, without PPARγ-associated adverse cardiac effects [190,191]. - Elafibranor, in a short-term trial (4–12 weeks), decreased ALT [190,192] - Elafibranor, in patients with biopsy-proven NASH, at a dose of 80 and 120 mg daily for 12 months, improved liver histology, liver enzymes, glucose and lipid profiles, and systemic inflammatory markers [193]. - Saroglitazar, an α/γ agonist, improved liver biochemistries and hepatic steatosis in a phase 2 study (NCT03061721) [194]
Pan-PPAR activator (Lanifibranor)	- A phase 2 trial (NCT03008070) is in progress to evaluate the effects of Lanifibranor, a pan α/δ/γ agonist.
Glucagon-like peptide (GLP)-1 and GLP-1 agonists (Liraglutide, Semaglutide, Tirzepatide, CotadutideDulaglutide, Exenatide, Albiglutide)	- GLP-1, acting as an insulin sensitizer, displays anti-NASH activity [195]. - Liraglutide (LIRA-NAFLD study), administered for 6 months in type 2 diabetic patients, induced weight reduction and a liver fat reduction of 31%, as assessed by magnetic resonance spectroscopy (NCT02721888) [196]. - Liraglutide has beneficial effects on liver enzymes [197]. - Liraglutide (LEAN Phase II trial) is effective in NASH patients with and without diabetes in inducing weight loss, resolution of steatohepatitis, and decreasing the progression of fibrosis, as compared with placebo. Potential gastrointestinal adverse effects: diarrhea, constipation, appetite loss (NCT01237119) [198]. - Semaglutide is currently tested in Phase II clinical trial. - Tirzepatide, a dual glucose-dependent insulinotropic polypeptide and GLP-1 receptor agonist, is under evaluation in patients with type 2 diabetes. Its efficacy and safety in NASH patients are currently being investigated (SYNERGY-NASH trial NCT04166773). - Cotadutide is a dual GLP-1 and glucagon receptor agonist studied in overweight subjects with T2DM with an effect on the decrease in aminotransferases levels (NCT03235050) [199].
Inhibitors of metabolic enzymes (Acetyl-CoA carboxylase [ACC] inhibitor; Firsocostat [GS-0976], PF-05221304, PF-06865571, PF-06835919)	- Firsocostat (GS-0976), a potent ACC inhibitor used in a clinical trial for 12 weeks, has been associated with significantly reduced hepatic steatosis and fibrosis marker TIM1 in patients with biopsy-proven NASH and F1–F3 fibrosis (NCT02856555) [200]. However, serum TG levels increased, possibly because of a compensatory increase in sterol regulatory element-binding protein 1 activity, with TG accumulation from peripheral FFA [201]. - PF-05221304 is a liver-directed ACC inhibitor and is being investigated in a phase 2 trial over 16 weeks in NAFLD patients (NCT03248882) [202,203]. - Notably, the inhibition of ACC reduces hepatocellular malonyl-CoA levels leading to increased mitochondria β-oxidation with a consequent decrease in PUFA and therefore improved liver steatosis [204]. - PF-06865571 is a diacylglycerol acyltransferase 2 (DGAT2) inhibitor. Although this agent might play a role in the clinical ground, no data are available so far. - PF-06835919 is an inhibitor of ketohexokinase (KHK, hepatic fructokinase), which is involved in the phosphorylation of fructose to fructose-1-phosphate. PF-06835919 might decrease steatosis in NAFLD patients (NCT03256526) [205]
Cleavage of citrate to generate oxaloacetate and acetyl-CoA (ATP-Citrate Lyase [ACLY])	- Excess nutrients activate ATP-citrate lyase (ACLY), which catalyzes the cleavage of citrate to generate oxaloacetate and acetyl-CoA. Could become a therapeutic target for the treatment of NASH [206]
Liver farnesoid X receptor (FXR) agonist—bile acid (Obeticolic acid [OCA])	- FXR is a bile acids nuclear receptor highly expressed in the liver and ileal mucosa. Activated FXR has a key role in the inhibition of lipogenesis and gluconeogenesis [26], restitution of insulin sensitivity, and suppression of bile acids synthesis [207]. - OCA (6-ethylchenodeoxycholic acid) is the lipophilic synthetic variant of the primary BA chenodeoxycholic acid (CDCA). Semi-synthetic agonist with 100-fold higher potency than CDCA. OCA promotes FFA oxidation and hepatic glycogen synthesis [27,208,209]. - In NAFLD, FXR is downregulated and can be activated by OCA [210]. - OCA at 25 mg/day orally for 72 weeks improved liver histology of NASH without worsening of fibrosis (45% of the treated patients vs. 21% in the placebo group). The liver enzymes serum alanine aminotransferase (ALT) and aspartate aminotransferase (AST) concentrations decreased during OCA treatment [27]. - In the FLINT trial, 23% of OCA-treated patients complained of pruritus, while its long-term safety and tolerability are still unclear. In some patients, OCA at 25 mg/daily caused an increase in low-density lipoprotein (LDL) cholesterol [27]. - The trial REGENERATE (NCT02548351) reports that patients on OCA 25 mg daily had resolution of NASH and no worsening of fibrosis at 18 months (when cases with F1 fibrosis were also included in the analysis) [211,212]. - The REVERSE trial (NCT03439254) in NASH-cirrhosis patients is in progress. - Response rate for OCA and other FXR agonists in NASH is about 25%, and this aspect points to the need for combination therapy with agents acting at different levels in NAFLD/NASH (See related paragraph).
FXR agonist—non-bile acids (Tropifexor, Cilofexor, EYP001Nidufexor, EDP-305)	- Tropifexor, in the FLIGHT-FXR phase 2 study (NCT02855164), was associated with a decrease in steatosis and a reduction in serum alanine aminotransferase and gamma-glutamyl transferase [213]. - Other agents under evaluation include Cilofexor (NCT03449446), EYP 001 (NCT03812029) and Nidufexor (NCT02913105) [204]. - EDP-305 (NCT03421431) has no/minimal cross-reactivity to TGR5 or other nuclear receptors. Improved pre-established liver injury and hepatic fibrosis in murine biliary and metabolic models of liver disease. Currently being tested in a phase 2 dose-ranging, randomized, double-blind, and placebo-controlled study evaluating the safety, tolerability, pharmacokinetics, and efficacy of EDP-305 in fibrotic liver diseases, including cholangiopathies and nonalcoholic steatohepatitis [214]
Enzyme inhibitors—Inhibition of stearoyl-CoA desaturase 1 (SCD1) (Arachidyl-amido cholanoic acid [aramchol])	- Aramchol, a fatty acid–bile acid conjugate, inhibits SCD1, an endoplasmic reticulum enzyme that catalyzes the rate-limiting step in the formation of monounsaturated fatty acids (MUFAs) from saturated FA, specifically oleate and palmitoleate from stearoyl-CoA and palmitoyl-CoA (the rate-limiting step in hepatocyte lipogenesis) [215]. - Aramchol was used in a phase IIa trial in 60 patients with biopsy-proven NAFLD for 3 months. This drug was safe, well-tolerated, and significantly reduced liver fat content (magnetic resonance spectroscopy). The anti-steatotic effect occurred in a dose-dependent manner with a trend of metabolic improvements. Effects on inflammation and fibrosis need further investigation [216,217]. - Aramcol was effective in a 52-week, phase 2b, placebo-controlled, randomized trial to decrease NASH fibrosis (NCT02279524) [218] and is being used in the phase 3/4 ARMOR clinical trial (NCT04104321).
Bile acids derivative (Norursodeoxycholic acid)	- Norursodeoxycholic acid has no effect on FXR. In adouble-blind, randomized, placebo-controlled phase 2 trial without histology, norursodeoxycholic acid induced a dose-dependent reduction in serum ALT (NCT03872921) [219]
Intestinal hormones (Fibroblast growth factor-19 [FGF-19]; Fibroblast growth factor-21 [FGF-21] and its analog Pegbelfermin)	- The enterokine FGF-19 is released when BA activates FXR in the terminal small intestine [26,220] and regulates energy expenditure [221]. FGF-19 acts on liver FXR via the FGFR4/β-Klotho receptor [26]. Human studies show that FGF-19 decreases hepatic fat and liver enzymes in patients with biopsy-confirmed NASH [222]. - FGF-21 originates from the liver, adipose tissue, and pancreas with effects on energy expenditure, improved insulin sensitivity, reduced sugar intake, and browning adipose tissue. FGF-21 is expressed mainly in the liver and is a potent activator of glucose uptake on adipocytes [223]. - FGF-21 displays insulin-sensitizing and antifibrotic effects in the liver. Tested in animal models and in a short-term trial in humans [224]. - Pegbelfermin is the pegylated FGF-21 analog acting on FGF-21 receptor beta (FGF21Rβ). Pegbelfermin, administered for 16 weeks, decreased hepatic fat fraction (by MRI proton density fat fraction) in a phase 2 study (NCT02413372) [225]. Further phase 2b clinical are FALCON 1 (NCT03486899) and FALCON 2 (NCT03486912), respectively, in patients with NASH with bridging fibrosis and in patients with NASH and compensated cirrhosis.
Hepatic thyroid hormone receptor (THR)-β-selective agonists (Resmetirom; VK2809)	- Resmetirom is a hepatic thyroid hormone receptor (THR)-β-selective agonist [145] with anti-steatogenic effects [226]. - Resmetiron, in the clinical trial MAESTRO-NASH, is being assessed in patients with NASH and stage 2 or 3 fibrosis (NCT03900429). - Resmetirom treatment for 12 weeks decreased the amount of liver fat by approximately 40%, with few adverse reactions [227]. - The effect of VK2809 in patients with NAFLD and hyperlipidemia is being investigated (VOYAGE trial, NCT04173065).
Sodium/glucose transport protein 2 (SGLT2) inhibitors (Empagliflozin, Canagliflozin, Dapagliflozin, Lipogliflozin)	- A moderate (3–4%) weight loss is documented with SGLT2 inhibitors, as well as a delay in the progression of kidney disease [228]. - In the liver, SGLT2 inhibitors increase the use of FFA [228]. - ALT levels decrease due to weight reduction and better glycemic control [229,230,231,232]. - Empagliflozin, dapagliflozin, and lipogliflozin use is associated with decreased intrahepatic fat content [230,233,234]
Immune response (Selonsertib [GS-4997])	- The innate immune response is involved in the pathogenesis of NASH, with pathways such as (ASC-1)-JNK, MAP kinase, ERK, and NFκB. Activated ASK1 induces downstream signaling transduction, leading to inflammation, apoptosis, and fibrosis [19,235]. Selonsertib is a selective inhibitor of ASK1, and short-term clinical trials exist in NASH patients [236]. - Experimental agents acting as ASK1 inhibitors are CASP8 and FADD-like apoptosis regulator (CFLAR) and TNF-a-induced protein (TNFAIP)3 [237,238]. - More studies required.
Chemokine inhibitors (CCR2/CCR5 receptor inhibitor Cenicriviroc)	- Cenicriviroc is an oral, dual CCR2/CCR5 receptor inhibitor, targeting C-C motif chemokine receptors 2 and 5 with effect on innate immunity, migration, and infiltration of inflammatory monocytes, macrophages, activation of stellate cells, and myofibroblasts leading to fibrosis. Studies are in progress on potential beneficial effects in NASH and inflammation [239], i.e., CENTAUR trial [240], and a phase-3 AURORA clinical trial (NCT03028740).
Deubiquitinase function (Cylindromatosis[CYLD])	- TAK1 belongs to the MAP3K family within the innate immunity signaling transduction (JNK–p38, NF-kB signaling pathways [241]). CYLD inhibits TAK1 activation through its deubiquitinase function (i.e., inhibits TAK1 overactivation without deletion) and might be effective in suppressing NASH progression [242]
Antifibrotic agents (ND-L02-s0201 anti-heat shock protein 47 [HSP47])	- The safety, tolerability, biological activity, and pharmacokinetics of ND-L02-s0201, a vitamin a-coupled lipid nanoparticle containing siRNA against HSP47, are currently under evaluation in subjects with moderate to extensive hepatic fibrosis (METAVIR F3-4) (ClinicalTrials.gov number: NCT02227459). The mechanism includes apoptosis of hepatic stellate cells.
Inhibitor of galectin (Belapectin)	- Galectin acts as a fibrogenic factor. In a subgroup analysis of patients without esophageal varices, 2 mg/kg belapectin did reduce hepatic vein portal gradient (HVPG) and the development of varices. ClinicalTrials.gov number: NCT02462967 [243].
Agent acting at extrahepatic levels (BAR502)	- Animal studies suggest that BAR502 is a molecule with dual activity as a ligand for FXR and TGR5 (a G protein-coupled receptor specific for BA). BAR502 leads to browning of white adipose in NASH [244]
Agents acting at extrahepatic levels (Probiotics)	- The intestinal microbiota and intestinal permeability appear to play a relevant role in NAFLD. Future therapies might therefore include the diagnosis of intestinal dysbiosis, as well as the use of single or combined probiotics [245]
Statin (Atorvastatin)	- Improvement of liver enzyme aminotransferase levels [246,247,248]
Fatty acids (Omega-3 fatty acids, Polyunsaturated fatty acids [PUFA])	- Beneficial effects in the animal model of NAFLD induced by a high-fat diet [249]. - Improvement in hepatic steatosis and aspartate aminotransferase levels [250,251]. - PUFA might contribute to decreasing the fat content in the hepatocyte [252] but had no effect in clinical studies evaluating the NASH activity score or fibrosis [253]. - The PUFA n-6 α-linoleic acid was able to protect hepatocytes from apoptosis via reduced c-Jun N-terminal kinase activation and mediators of inflammation [253].
Antinflammatory agent (Aspirin)	- Reduced fibrosis and evolution to NASH [254].
Natural pentacyclic isoquinoline alkaloid (Berberine)	- Hypolipidemic effect, improvement of liver fat, body weight, HOMA-IR. Improves OXPHOS in the liver of HFD-fed rats and increases mitochondrial SIRT3 activity [255]. - Beneficial effects in NAFLD [256]. - Currently being tested in a multicenter, double-blinded, randomized, placebo-controlled clinical trial in subjects with nonalcoholic steatohepatitis (NASH) treated for 48 weeks. ClinicalTrials.gov identifier: NCT03198572.
Inhibitor of mitochondrial pyruvate carrier (MSDC-0602K)	- Evaluated in a 52-week, phase 2b dose-ranging clinical trial in subjects with biopsy-proven NASH, MSDC-0602K use was associated with significant reductions in glucose, glycated hemoglobin (HbA1c), insulin, liver enzymes, and NAFLD Activity Score (NAS) vs. placebo (NCT02784444) [189]. - A phase 3 clinical trial is planned in patients with TD2M and NASH (NCT03970031).

**Table 4:** Therapeutic strategies to ameliorate mitochondrial function in NAFLD.

General Measures	Notes
Lifestyles	Moderately hypocaloric diet plus physical exercise might improve mitochondrial function and alleviate inflammation [259,260,261]
Antidiabetic drugs	Elafibranor [190,193]
	Liraglutide [262]
	Metformin [263]
	Thiazolidinediones (pioglitazone) [264], MSDC-0602K [189]
Bile acids	Obeticholic acid [27,208,209]
	Ursodeoxycholic acid [265]
Agents acting as antioxidants, on nuclear receptors or mitochondrial metabolism	Vitamin E (α-Tocopherol) [64]
	Tempol [266] ^1^
	Resveratrol [267,268,269,270] ^1^
	Mitoquinone (Mito-Q) and Mitovitamin E (MitoVit-E) [271,272,273] ^1,2^
	Silymarin (major component is Silybin) [108,274,275]
	Corilagin [276] ^2^
	Anthocyanins (i.e., Cyanidin) [277,278] ^1^
	Dihydromyricetin [279] ^1^
	Berberine [255] ^1^
	Hydroxytyrosol [249] ^1^
	Cysteamine [280,281]
	Pentoxifilline [282,283,284]
	Avocado oil [285,286,287] ^1^
	Pegbelfermin (via FGF21R beta) [225]
Mitotherapy	Exogenous mitochondria tagged with green-fluorescence protein (GFP) and retrieved in mouse liver, lungs, brain, muscle, and kidneys [288,289] ^1^. Improved energy production may restore hepatocyte function [290] ^1^
Miscellanea	Aramchol [216,217]
	Baicalin [291] ^1,2^
	Nitro-oleic acid [292] ^1^
	Carboxyatractyloside [293] ^1^
	Genistein [294]
	Firsocostat (acetyl-CoA carboxylase (ACC) inhibitor) [295]

^1^ Further evidence is required (animal/in vivo evidence); ^2^ further evidence is required (in vitro study).
